# The Elderly’s Independent Living in Smart Homes: A Characterization of Activities and Sensing Infrastructure Survey to Facilitate Services Development

**DOI:** 10.3390/s150511312

**Published:** 2015-05-14

**Authors:** Qin Ni, Ana Belén García Hernando, Iván Pau de la Cruz

**Affiliations:** Departamento de Ingeniería Telemática y Electrónica, Universidad Politécnica de Madrid, Carretera de Valencia km. 7, Madrid 28031, Spain; E-Mails: anabelen.garcia@upm.es (A.B.G.H); ivan.pau@upm.es (I.P.C.)

**Keywords:** activity monitoring, activity characterization, sensors, data processing, smart home, independent living, e-health services development

## Abstract

Human activity detection within smart homes is one of the basis of unobtrusive wellness monitoring of a rapidly aging population in developed countries. Most works in this area use the concept of “activity” as the building block with which to construct applications such as healthcare monitoring or ambient assisted living. The process of identifying a specific activity encompasses the selection of the appropriate set of sensors, the correct preprocessing of their provided raw data and the learning/reasoning using this information. If the selection of the sensors and the data processing methods are wrongly performed, the whole activity detection process may fail, leading to the consequent failure of the whole application. Related to this, the main contributions of this review are the following: first, we propose a classification of the main activities considered in smart home scenarios which are targeted to older people’s independent living, as well as their characterization and formalized context representation; second, we perform a classification of sensors and data processing methods that are suitable for the detection of the aforementioned activities. Our aim is to help researchers and developers in these lower-level technical aspects that are nevertheless fundamental for the success of the complete application.

## 1. Introduction

Recent advances in sensing, networking and ambient intelligence technologies have resulted in a rapid emergence of smart environments. Among these, the so-called Smart Home (SH) has gained a lot of attention for the provision of enhanced quality of life within the home. The smart home concept was formalized by Lutolf [1], who primarily focused on the integration of different services within a home environment by using a common communication system. More recently, Satpathy [2] proposed a smart home concept more centered on helping the residents live independently and comfortably with the help of mechanical and digital devices. This definition is closer to our current understanding of SHs.

One of the motivations for smart home research is the significant worldwide increase of an aging population. In fact, according to the World Health Organization (WHO), the world’s elderly population (defined as people aged 60 and older) has increased drastically in the past decades and will reach about 2 billion in 2050 [3]. In Europe, the proportion of the EU27 elderly population above 65 years of age is foreseen to rise to 30% in 2060 [4].

The elderly have specific health issues that have to be considered. A significant proportion of elderly population suffer or may suffer with higher probability from age-related conditions such as Parkinson’s disease, diabetes, cardiovascular disease, Alzheimer’s disease, different chronic diseases and limitations in physical functions. For them, SH technologies may help to enhance quality of life, prolong independent living and reduce caregivers’ necessary time and healthcare costs in general, without losing the safety that a continuous and unobtrusive monitoring provides. Thus the benefits of these technologies are not only for the older adults, but also their families, caregivers and society in general. This is what is sometimes known as Ambient Assisted Living (AAL).

Research objectives in this area range from low-level data acquisition by sensors up to high-level data integration and inference of knowledge through both data-driven and knowledge-driven approaches. Many recent works are related to activity recognition as a means of extracting higher-level information. There are different types of activities, but the common ground to all of them is that they should be recognizable as such by a non-technician (e.g., “preparing a meal”, “taking a bath” or “watch television while sitting on the sofa”). If human activities are correctly and automatically identified, a wide range of applications and services become possible, such as detection of health emergencies, early disease detection, professional advice on routine lifestyle, health status monitoring and help in treatment prescription. Some concrete examples of applications can be found in [5] (a mobile emergency response system), [6] (a fall detection system) and [7] (which deals with monitoring activities of daily life and recommending services for active lifestyle).

**Figure 1:**
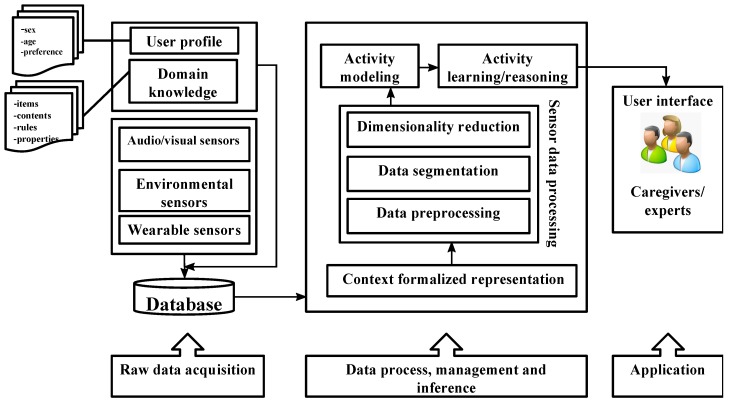
Overall architecture of activity monitoring systems in smart home environments.

Figure 1 shows the general structure of activity-based AAL systems. Three stages are identified: raw-data acquisition, sensor data processing to obtain context information and learning/reasoning methods to identify activities and provide caregivers and experts with useful and significant information.

It is clear that in order for activities to be detected, a set of sensors have to be deployed inside the home. Some of these sensors may be located on furniture, appliances, walls and doors, whereas others may be “worn” by the inhabitants, attached to their bodies either directly or indirectly (e.g., a smartphone that is carried in a pocket). In general, different sensors are needed simultaneously to correctly identify the different activities, and each of them may provide its measurements (*i.e.*, the raw data) in a different format. Raw data by itself is therefore of little (or no) use for the activity detection algorithms. The preprocessing of this data is a must in order to obtain useful and significant information for the application.

Whereas it is possible to find a good number of research reviews related to aspects such as sensor design, monitoring techniques or machine learning algorithms and reasoning approaches, to the best of our knowledge the underlying and fundamental issues of activity context information representation, proper sensor selection and sensor raw data processing have not received enough attention yet in the concrete context of elderly people needs. In addition, reviews tend to focus on describing solutions from a technical viewpoint, but not so much from the perspective of service developers and providers. We devote the whole Section 2.1 (Recent surveys on smart homes) to summarize some of the most significant and recent research review papers in this area, in order to contextualize the principal open issues.

The main objective of this review paper is twofold: first, we propose a classification of the main activities considered in smart home scenarios targeted to the elderly’s independent living, as well as their characterization and formalized context representation; second, we advance towards a general set of guidelines that would help researchers and developers select the sensors and processing techniques best suited to the target activities to detect, focusing on older adults and indoor smart home activities. Some processing techniques (especially preprocessing and segmentation approaches) are identified and classified in detail. Finally, we present some identified research challenges and suggestions for further research.

The rest of the paper is organized as follows: Section 2 summarizes the most recent smart home projects and applications, establishing a classification into three categories that are relevant for older adults’ independent living. Also in this context, a classification of the main activities considered in smart home scenarios and their characterization and formalized context representation is proposed in Section 3. A review of the different types of sensors used to monitor these activities as well as their main utility and limitations is provided in Section 4. Section 5 is devoted to sensor data processing techniques, together with their applicability to different scenarios related to the elderly’s independent living, whereas Section 6 contains the description of some identified research challenges and possible future directions in this area. Finally, we conclude this review in Section 7.

## 2. Smart Home Projects and Applications

The numerous research advancements achieved in the SH area have improved access to healthcare services for the elderly. Various projects and applications have been developed throughout the world to prolong independent living for this population segment. In this section, we review the most recent surveys on SH, as well as the projects and applications tailored to the elderly’s specific requirements.

Even if this paper is not intended to dive deep into the complex matter of human factors, we cannot ignore the fact that these are increasingly gaining importance in any information and communication technologies (ICT) system. This is why we will conclude Section 2 with a brief summary on the importance that human factors have for the success and acceptability of any SH deployment.

### 2.1. Recent Surveys on Smart Homes

A recent survey on smart homes, written by Alam *et al.* [8], gives a general overview of past developments, present situations and future challenges. It reviews the smart home projects according to their research objectives and three desired services: comfort, healthcare and security, and also describes information about sensors, multimedia devices, communication protocols, and algorithms used for these desired services. Another survey on ambient assisted living technologies for older adults is conducted by Rashidi and Mihailidis [9], who summarized AAL technologies and tools in terms of smart homes, assistive robotics, e-textile and wearable sensors, also exploring healthcare applications that focus on activity recognition algorithms and context modelling.

Salih *et al.* [10] presented a review on ambient intelligence assisted healthcare monitoring, summarizing wireless sensor networks technologies, communication technologies and applications. Their research mainly focuses on ambient intelligence methods and data mining techniques used in wearable and ambient sensor monitoring within smart home for older adults and patients with chronic diseases.

Peetoom *et al.* [11] conducted a systematic investigation on current literature on monitoring technologies to detect activities of daily life or significant events (e.g., falls and changes in health status) for elderly people in-home. Their research identifies five main types of monitoring technologies: PIR motion sensors, body-worn sensors, pressure sensors, video monitoring and sound recognition. Additionally, the functionalities and outcomes of applying these technologies to prolong independent living of elderly people are demonstrated. The results of their studies suggest positive effects, both to residents and caregivers, of using monitoring technologies.

Khusainov *et al.* [12] presented a study on automated methods for real-time human wellness monitoring and various algorithmic techniques on sensor data analysis to devise effective means of addressing the demands of assisted living and clinical observation. Their survey reviews three areas of sensor-based monitoring systems: sensor types, frameworks and applications; data collection, processing and analysis; and research gaps, limitations and challenges. 

Our review differs from the above in two significant aspects: firstly, we focus on activities and applications useful for the elderly inside a SH, which guides the application taxonomy we use, and furthermore, we propose a formalization of the description of the activities including properties and context representation. Secondly, although our review does not include aspects such as hardware and communications, it contains a deeper discussion and classification on signal processing techniques (especially preprocessing and segmentation approaches).

Avci *et al.* [13] presented a survey about activity recognition for healthcare, wellbeing and sports applications by using inertial sensors. This work is arranged according to the main steps involved in the activity recognition process and the main techniques utilized in each of those. However, this survey only deals with inertial sensors. Bulling *et al.* [14] give a comprehensive introduction on human activity recognition, but similarly, they focus on a specific type of sensors (in this case body-worn inertial sensors).

### 2.2. Smart Home Projects

In this section, we conduct an investigation on the most significant smart home projects that aim at enhancing assisted living for older people in the recent past. These SH projects fully simulate the smart home environment including the deployment of a wide range of sensors. A chronological order is chosen for this presentation, in order to give a clearer picture on how the expectations and research issues related to SH have evolved with time. In the earliest projects presented, the main issues discussed were related to physical and logical connectivity of devices. This kind of projects evolved to multidisciplinary approaches very focused on improving the usability of the interaction of devices with the inhabitants. This form of interaction demanded technologies with a high grade of abstraction such as those related to Artificial Intelligence which, in turn, has fostered the evolution to more natural and personalized interaction approaches. In addition, these technologies introduced the capacity of managing uncertainty, a common issue in smart home solutions. In recent years the projects have no longer been focused on the Smart Home as a final objective but rather as an enabling technology to achieve other purposes such as independent living for seniors.

A summary on the widely reusable datasets collected by the different smart home projects is also presented to make choosing between them easier for researchers. 

GatorTech [15] is an early smart home project carried out at the University of Florida, which integrated a set of sensors and devices to provide services such as voice recognition and inhabitants’ activity tracking. Outstanding early smart home projects also include the following: Adaptive Versatile home (MavHome) [16] from the University of Texas at Arlington, PlaceLab [17] from the MIT and Intelligent System Lab (ISL) [18] from the University of Amsterdam.

The CASAS smart home project [19], developed at Washington State University in 2007, is a multi-disciplinary research project focused on creating an intelligent home environment by using unobtrusive sensors and actuators. The research areas included in CASAS are assistive technology, artificial intelligence, machine learning and activity recognition. This same team has developed in its recent research the “smart home in a box” [20], which is a lightweight smart home design that is easy to install and provides SH capabilities out of the box with no customization or training needed. These capabilities include activity recognition, which provides real time activity labelling as sensor events arrive in a stream, and activity discovery for unlabeled data by using an unsupervised learning algorithm.

SWEET-HOME [21] is a nationally supported French research project that aims to design a new smart home system based on audio technology. This project has three main goals: providing an audio-based interaction technology that lets the users have full control over their home environment, detecting distress situations and easing the social inclusion of the elderly and frail population. An interesting research direction of their smart home system is the context-aware decision process, which uses a dedicated Markov Logic Network approach to enhance the ability of coping with uncertain events inferred from real sensor data [22].

A recent smart home project is “Unobtrusive Smart Environments for Independent Living” (USEFIL), an FP7 project which started in 2011. It aims to provide an advanced and affordable health-care assistance in a smart home environment, adopting a three-layer architecture and an unobtrusive sensor network to support a Decision Support System (DSS) for providing inputs to monitoring apps by user-friendly interaction [23]. A limited set of sensors and devices, such as a wrist wearable unit, a camera, a microphone and a Kinect sensor, are used in a SH setting to identify the basic physical activities (lying, sitting, walking, standing, cycling, running, ascending and descending stairs) of elderly people [24]. A low cost off-the-self system and open source platforms are developed to facilitate the generation of applications addressing the gap between advanced technologies and aging population.

Qing and Mohan proposed a Smarter and Safer Home solution at CSIRO to enhance elderly people’s quality of life [25]. To achieve this, a number of environmental sensors are placed in various locations within smart home, acting as non-intrusive monitoring devices for identifying human behaviors. Based on this project, Zhang *et al.* [26] proposed a Smart Assistive Living (SAL) platform to enable elderly people to remain at their homes independently as long as possible. Sensors placed in smart home are expected to provide a continuous data stream to a server. Extracting and analyzing these data using machine learning mechanisms are helpful to perform diagnosis and decision making by clinical experts and health caregivers.

These smart home projects provide a large amount of datasets, some of which are publicly available and can be used by researchers to conduct further studies. Among the publicly available datasets listed in the “Home Dataset” [27], those collected by MIT, the University of Amsterdam and Washington State University are widely used in Smart Home research. Moreover, the dataset provided by MIT [17] with more than 900 sensor inputs, including those coming from motion, switch and RFID sensors, is, to the best of our knowledge, by far the largest dataset collected from a real-world environment. In addition to data from embedded sensors, the dataset provided by [28] contains acceleration and gesture data. The benchmark dataset described in [29] is also widely used and contains data collected from a set of nine inertial sensors attached to different parts of the body. Concretely, there is motion data related to 33 fitness activities recorded from 17 volunteers.

### 2.3. Smart Home Applications Suited to Elderly People

There is not a single way of classifying the SH applications that may enhance older adults’ quality of life and health status. We propose here a division into three main categories and summarize some of the most significant recent work found in the literature inside each of them. These categories are: “Specific health monitoring”, “Daily activities monitoring, prediction and reminding”, and “Detection of anomalous situations”. We also note that these categories are not mutually exclusive: the same application may contain ingredients that fit into more than one of them.

#### 2.3.1. Specific Health Monitoring

On occasions older adults need specific monitoring of either vital signs or daily activities in order to assess if a medical condition is being correctly controlled. Other possible aims of this application are to detect early when an illness is developing or to react quickly when a sudden change in medical parameters occurs. In the following we summarize information from some projects focused on this type of functionality and related with specific diseases such as diabetes and stroke patients.

Pulkkinen *et al.* [30] described a platform for monitoring daily activities by using different types of sensors for elderly people with diabetes at home and help detecting if the patients are following the recommended exercise and diet routines. Data obtained from the sensors for a long period of time can be used to distinguish the user’s life patterns, helping doctors to design treatment plans and, after that, providing the patient with automatic notifications. Chatterjee *et al.* [31] built an in-home activity monitoring system to detect the daily routines of elderly people with diabetes by using environmental sensors and body wearable sensors. Daily messages based on previous behaviors, a tailored health newsletter that summarizes biological parameters and blood glucose level prediction are sent to the users and caregivers to determine the health status.

Chiang *et al.* [32] developed a set of wireless sensor network devices to support physical therapy to be carried out by elderly stroke patients in smart home environments. Stroke patients usually have to repeat specific movements or postures during their rehabilitation, as indicated by their physiotherapists. Thus, the system described by Chiang *et al.* is aimed at measuring both static postures and dynamic movements, and to record them as routines. As an example of vital signs monitoring, Sardini and Serpelloni [33] developed a t-shirt with embedded sensors to take measurements that include heart rate, respiratory rate and body temperature of the patient. The acquired data can be wirelessly sent to caregivers and then analyzed continuously by healthcare experts to perform proper evaluation and assess sudden changes in the health status of the patient. 

Apart from health monitoring systems developed in laboratory environments, some commercially available solutions also exist. Those solutions have a broader scope in healthcare management but are usually less integrated with the home. To mention a few, Genesis by Honeywell [34], TeleStation by Philips [35] and HealthBuddy by Bosch [36] fall into this category.

#### 2.3.2. Daily Activities Monitoring, Prediction and Reminding

Patterns obtained from daily routines can be very useful for helping elderly people who suffer from any kind of cognition decline. Reasoning over these patterns allows to predict actions and to provide reminders of the things that should be done (for instance, guidance when the person does not remember the steps to follow to complete an activity). Many elderly people who live alone also suffer from other age-related diseases such as depression and diabetes, and the activities of these patients can be influenced by their diseases. For example, patients with depression could exhibit infrequent leaving the house, less talking, sleeping disorder and less eating, whereas patients with diabetes could perform frequent drinking, eating, sleeping and toileting. Being capable of detecting these patterns can help in diagnosing these illnesses.

In the literature different approaches can be found when dealing with activities detection: detection of activities as the main objective, remainder of tasks to inhabitants, detection of changes in routines as signs of possible risks or diagnosis of specific diseases based on activity patterns. Some significant examples of each of these approaches are discussed in the following.

Chernbumroong *et al.* [37] proposed a practical multi-sensor activity recognition system for monitoring daily life activities, including brushing, exercising, feeding, ironing, reading, scrubbing, walking, walking on stairs, washing, watching and wiping by using seven types of sensors attached to the body.

Lara *et al.* [38] proposed Centinela, a system that continuously monitors five activities (walking, running, sitting, ascending and descending) by using a single sensing device and a mobile phone. The system consists of a portable and unobtrusive data collection platform to provide real-time human activity monitoring. The mobile application they implemented is based on acceleration and physiological signals to recognize physical activities automatically.

Chaminda *et al.* [39] proposed a smart reminder system for reminding older adults with memory impairment of the forgotten complex activities in smart home environment. Reminders of forgotten activities are predicted according to the inhabitant’s current location, current behavior patterns and past activity patterns. Wearable sensors are used to acquire data to generate these activity patterns. A similar system is proposed in [40]. Suryadevara *et al.* [41] develop an intelligent home monitoring system to detect behavior changes and forecast the behavior of elderly people.

Han *et al.* [42] proposed a four layers healthcare framework to predict the risk of depression and diabetes by monitoring long-term disease related activity and generating long-term activity patterns. When the symptoms of these diseases appear in people with irregular activity patterns, the information will be sent to doctors and caregivers for early detection and prevention of the depression and diabetes.

#### 2.3.3. Detection of Anomalous Situations

Anomaly detection aims to identify uncommon patterns in a dataset, that is, patterns which do not conform to the notion of normal behavior [43]. To detect them, techniques based on rule-based approaches, temporal relation discovery approaches and similarity-based approaches may be used. The identification of anomaly patterns, especially in daily activities monitoring systems, can be significantly valuable for experts to make decisions or diagnoses in emergency situations. It can also be used to provide elderly people with memory impairments with reminders or audio signals if an anomalous behavior is being detected.

As an important subset of the detection of anomalous situations we find fall detection applications. Older adults are more prone to falls, and their consequences are in general more serious than for younger people. This is the reason why it is possible to encounter a good number of proposals specifically focused in fall detection for the elderly, as an important issue to facilitate their independence at home.

In [44], Phua *et al.* proposed an Erroneous-Plan Recognition (EPR) to detect faults in the daily activities of elderly people with dementia, by using sensors deployed in the SH to monitor daily activities. When an error is detected, timely audio or visual prompts are sent to the dementia patients to replace some of their diminished memory and enhance their problem-solving abilities. Similar works about anomaly detection for dementia patients can be found in [45,46,47].

Ordóñez *et al.* [48] developed an automated behavior analysis system for elderly people who live alone at home. It captures measurements from various sensors, detects the activities of each user, and is capable of detecting anomalous behavior that reflects changes in health status, by learning standard behavior patterns. Gjoreski *et al.* [49] proposed a system to monitor users’ daily activity by combining two accelerometers and an electrocardiogram (ECG) sensor. Measured acceleration data can be analyzed in conjunction with the ECG signals to detect anomalies in the user’s behavior and heart-related problems.

Regarding fall detection systems, Wu and Xue [50] developed a portable pre-impact fall detection and injury prevention system with inertial sensors. By using the inertial frame velocity profile of the body, they distinguish falls from non-fall activities as well as identify early falls by using a threshold detection algorithm. The designed pre-impact detection device is embedded into a wearable belt that can be worn by the older adult when they are performing daily activities. This system has been proved to increase the confidence of independent living for elderly people.

Rimminen *et al.* [51] also proposed a fall detection system by using a near-field imaging floor sensor and pattern recognition. The shape, size and magnitude of the patterns are used for classification, and the result shows good performance on fall detection even on unclear falls (ending up sitting or on one’s knees) and a good tolerance of daily activities. Zhuang *et al.* [52] described a fall detection system to distinguish noise coming from falls from other noise in smart home environment. In their system they only use a far-field microphone to identify various sounds. Then a Gaussian Mixture Models (GMM) supervector is used to model each fall or noise segment by applying Euclidean distance to measure the pairwise difference between audio segments. A Support Vector Machine built on a GMM supervector kernel is used to classify audio segments into falls and various types of noise.

More generally, although also usable for detecting falls, Ghasemzadeh *et al.* [53] introduced a physiological monitoring system, which collects acceleration and muscle activity signals to assess standing balance. They use machine learning algorithms and statistical techniques to infer relevant information from the correspondence between the accelerometer and the electromyogram (EMG) sensors measurements.

### 2.4. Human Factors

Few applications and systems are as sensitive to human factors as the ones with which this paper deals. Generally speaking, the consideration of human factors in technological systems consists in integrating, from the very early stages of the design, the specific capabilities, limitations and preferences of the human beings that are going to be involved with the application. In the case of Smart Home applications related to the elderly (or any other population group), acceptability is probably the most important factor to consider, not only for the older people themselves but also for their caregivers. Some authors have identified a friendly design which includes these human factors as an important element to improve the acceptance of the deployed solutions [54].

Design patterns are design solutions that have proven to be effective in some specific area or for a specific set of functionalities. Upon completion of this survey we have not found in the literature any design pattern oriented to encourage the acceptance of solutions for SH. However, users’ acceptance of solutions should be a prerequisite for proper implementation. In fact, according to Gaul *et al.* [55], the level of acceptance of technological solutions in the medical field is low, especially in the case of SH solutions oriented to the elderly. This same paper states that existing solutions are technically viable but lack a proper study of human factors, leading to acceptability problems. There are generic guidelines that establish design principles for applying human factors in sensitive areas such as telecare [56]. However, these guidelines do not provide design aids to develop solutions aligned with the factors described.

Even if general design patterns have not yet been proposed related to human factors in SH, it is fair to mention that there are authors who have considered these issues into their software architectures to a greater or lesser extent. For instance, Becker [57] performed a detailed review of the quality requirements that AAL environments must comply with, as well as their related technologies and architectural models. The conceptualization found in this paper is useful when dealing with the software architectural requirements, although it is not easily transferable to a low-level design made by developers. For their part, Vega *et al.* [58] identified the main challenges in the development of services based on smart environments (considered as a generalization of SH) and proposed an architecture to deal with these. The proposed architecture is based on the model-view controller paradigm commonly used in programming. Unlike other approaches, the core functionality is fully specified in the documents exchanged between the entities of the architecture, called Document-Contracts. This architecture facilitates the development, maintenance and creation of new solutions for non-technical users because the behavior of the system will be guided by the definition of these Document-Contracts in a high level language. It can also be adapted to the specific technical conditions of each home without altering the basic functionality of activities. However, the authors of [58] do not specify any design pattern to be used in the definition of the activities.

## 3. Conceptualization and Formalization of Activities in Smart Home

A clear understanding of the target activities in SH and their specific characteristics is critical for proper sensor selection and system design. In addition, accurate knowledge on the activities’ context, such as location and involved objects, if included into the activity conceptualization, may enhance the effectiveness of SH systems. In this section we firstly describe a taxonomy of SH activities in a manner that is meaningful for our target population (the elderly). This leads to a proposal for the formalization of activities, including their relationships with their context as well as with other activities. We then go into deeper detail on the specific techniques and languages that have been proposed in the literature for describing contextual information in a formalized manner. The whole section organized in this manner presents information that grows in specificity and technical detail with each sub-section, paving the way for Section 4, in which sensors capable of capturing the relevant information are tackled.

### 3.1. Taxonomy of Activities

In this sub-section, we propose a taxonomy applicable to activities that are related to healthcare, wellbeing and independence in SH environments, with special emphasis on the elderly population. This sub-section includes information of a very general and high-level nature, appropriate for being significant and understandable to personnel which is expert in the interpretation of the medical and care implications of specific activities (e.g., doctors) but not in the technical low-level aspects of the SH deployment.

On one hand, Activities of Daily Life (ADLs) are defined as “the things we normally do in daily living including any daily activity we perform for self-care such as feeding ourselves, bathing, dressing, grooming work, homemaking and leisure” [59] by the medical community. ADLs are used in smart home as an umbrella term encompassing self-care and domestic activities or tasks that the user undertakes routinely. The capacity of performing ADLs at home without assistance from other people can be considered as a reference for the estimation of the independent living level of the older adults [60]. Katz and Lawton define a range of ADLs [61] and Instrumental ADLs (IADLs) [62]. According to their definitions, the ADLs can be subdivided into personal self-care activities, or basic ADLs (BADLs), and domestic activities, or instrumental ADLs (IADLs). BADLs (a concept commonly used in current literature related to SH, see for instance [63]) refer to the necessary self-care activities, such as bathing, dressing, eating and drinking, functional mobility, using toilet and grooming. IADLs are activities which are not strictly necessary but let an individual live independently in a community, for instance shopping, housekeeping, managing money, preparing food, taking medication, using telephone and transportation. 

On the other hand, ambulatory activities are related to either specific motions or postures of the person. These activities can be subdivided into stationary activities, transitional activities and dynamic activities. Stationary activities describe a posture such as sitting, standing or lying. Transitional activities refer to the change from a stationary state to a dynamic state or vice versa, such as sit-to-stand, stand-to-sit, stand-to-walk and sit-to-lie. Dynamic activities include a set of simple dynamic actions such as walking, running, cycling and jogging. Ambulatory activity monitoring is very useful for detecting the physical activity level, promoting health (e.g., doing exercise) and detecting hazardous situations such as falling. Additionally, ambulatory activities can be useful for estimating the psychological wellbeing of older adults. For example, Tartarisco *et al.* [64] proposed a system for prolonged stress monitoring during normal activity performance in a smart home environment. They evaluated the individual immediate stress level by analyzing both the ambulatory activity frequency and the heart rate when the individual performs these ambulatory activities.

The classification and detailed description of the activities that are significant for the elderly well-being and independent living in SH is included in Table 1.

**Table 1:** Taxonomy of activities significant for the older adults in SH.

Type	Activity	Description of How the Activity Relates to the Elderly Independent Living
Basic ADLs	Bathing	Performs sponge bathing, tub bathing or showering without assistance
	Brushing teeth	Brushes one’s teeth without assistance (including the use of toothbrush and toothpaste)
	Dressing	Puts on and off clothes and shoes without assistance (except for tying shoes)
	Using toilet	Goes to toilet, uses it, dresses and returns without assistance (may use cane or walker)
	Eating and drinking	Feeds oneself and drinks without assistance (including the use of cutlery)
	Sleeping	Sleeps on a bed in the bedroom without assistance
Instrumented ADLs	Preparing meals	Chooses material and food in the kitchen, prepares meals autonomously without assistance
	Preparing drinks	Chooses the type of drinks, prepares drinks with sugar or milk
	Resting	Reads a book, listens to music, operates and watches TV without assistance during leisure time
	Housekeeping	Keeps house clean (sweeps floor with broom, washes dishes and glasses in kitchen, *etc.*) and does housework (such as ironing) without assistance
	Using a telephone	Picks up the telephone, dials the number, has a conversation or answers a call without assistance
	Taking medicine	Takes the prescribed medicines appropriately and timely without assistance
Ambulatory Activities	Walking	Walks from one place to another, walks up or down the stairs without assistance
	Doing exercise	Does exercise such as running and cycling without assistance
	Transitional activities	Performs transitional movements (such as sit-to-stand, sit-to-lie, stand-to-sit, lie-to-sit) in and out of bed or chair without assistance
	Stationary activities	Sits in the sofa, stands for a period of time (may use cane or walker), lies in bed or sofa

To compile the information contained in Table 1 we have taken into account the contributions included in the works referenced above. This taxonomy establishes a first basis for the activities definition that is aimed at decoupling the specification of sensors and processing techniques from the final service high level description. Depending on the application specific goals, some activities will be more interesting to be detected than others. For instance, a healthcare application for elderly people may be concerned about the user’s movements and fall detection, but an assistance application for elderly people with cognitive decline would be more focused on the sequences of activities that the user performs.

In general, in smart home applications it is critical to detect the boundaries of an activity and then identify what the concrete activity carried out is. This can be achieved with a proper characterization of the activities. As a first step towards this, we propose the details of a conceptualization of activities and their context in the following sections.

### 3.2. Activity Conceptualization

Once a first taxonomy has been described, the next step for the characterization of activities is related to their conceptualization. The conceptualization process allows a first characterization of the activities in terms that can be later formalized to be understandable by computers. Thus, it is a step forward towards a completely detailed and automated treatment of activities. 

**Figure 2:**
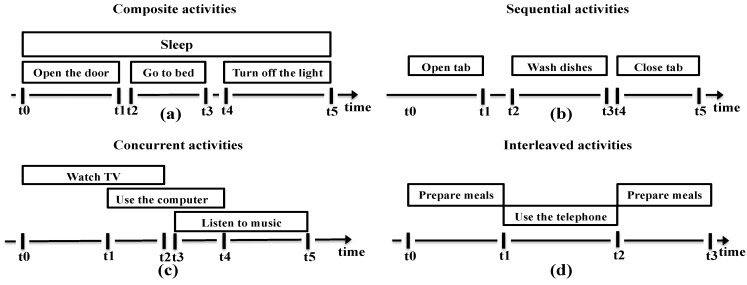
Diagrams showing different relationships between activities: (**a**) composite activities; (**b**) sequential activities; (**c**) concurrent activities; (**d**) interleaved activities.

Since an activity is usually related with other activities, the first classification we use for the conceptualization deals with the relationships among different activities. These relationships can be mainly classified into the following categories, depending on the interactions that take place during the activity timespan:

1. *Specialization of activities*—Activities can be categorized at multiple granularity levels. In [65], the authors distinguish the ADLs in the smart home from the actions, in order to define the human behavior at different complexity levels and durations. An action is an atomic activity that is performed by a single subject and lasts for a relatively short time. Some examples of action are “open the door”, “turn on the light” and “go to bed”. An ADL is usually defined as a more complex behavior performed by either a single user or multiple users and which lasts for a longer time than an action. Furthermore, a so-called coarse-grained activity may be specialized into two or more fine-grained activities. For example, “preparing drinks” may have as its child activities: “preparing hot drink” and “preparing cold drink”, whereas “preparing hot drink” can be further broken down into its child activities: “preparing tea”, “preparing hot milk” and “preparing coffee”. Meditskos *et al.* [66] defined the specialization of activities differently. As an example, in their activity pattern, the activity “night sleep” is defined as the overall night sleep activity of a person and the activity “out of bed” is detected when the person gets out of bed. With the addition of context description and activity type interpretation, an “out of bed” activity can be further specialized as a “bed exit” activity, which refers to the “out of bed” activity when it occurs during the “night sleep”.
2. *Composition of activities—*Most complex ADLs are composed of an ordered succession of simpler activities. For instance, “sleeping” may consist of: “opening the door”, “going to bed”, and “turning off the light” (as shown in Figure 2). The ordering of the simple activities may depend an individual’s preferences or habits, thus leading to several variants of an activity. Furthermore, activities may have time-related connections to each other, to form a composite activity. At this respect we may distinguish three situations: sequential activities, concurrent activities and interleaved activities. Figure 2 shows graphically these temporal relationships.

In terms of time interval, the so-called Allen relations [67] are commonly used to describe temporal links between activities: (1) Sequential activities are described by associating their time intervals using the before/after and meet/meetby relations. The before/after relation is found when one activity is performed either before or after another activity, and the two associated time intervals have a gap between them. For example, “opening tab” occurs before “washing dishes” and “closing tab” occurs after “washing dishes”. On the other side, the meet/meetby relation indicates that the two associated time intervals do not have a gap between them; (2) Concurrent activities occur at the same time, thus they share the same time intervals either fully or partially. There are nine options in this case: the overlaps/overlappedby relation indicates that the two intervals have a common shared sub-interval, and one interval starts or finishes before the other; the contains/during relation is found when the interval of a composite activity encloses the interval of its composing activities; the starts/startedby and finishes/finishedby relations relate to the case of an activity that starts or finishes during the timespan of another activity with longer time interval; the equals relation indicates that two activities occur in parallel and thus their start and finish intervals occur at the same time [51]. Concurrent activities take place either when one user performs two different activities simultaneously or when multiple users perform activities at the same time. For example, one user can perform “watching TV” and “using the computer” at the same time, or two users can perform “drinking tea” together; (3) Interleaved activities have time intervals that “preempt” each other, which indicates that a long and complex activity has a long time interval that contains the shorter one. The during/contains relation is also used to describe interleaved activities, but with a different meaning to the concurrent activities, since in this case the two interleaved activities are not part of the same coarse-grained activity. For example, the user may use the telephone or drink water at intervals that occur during the activity “prepare meals”.

Besides the relationships between activities, the understanding of context information of an activity is also critical in activity monitoring. Within a smart home environment, there exist many types of contextual information that can be used to characterize an activity, such as interaction with objects, location and time. Object and location are concepts used very much to describe and distinguish activities. Object refers to the relevant participant in an activity, either a person or a physical object. For example, if the TV set is the object, it can be inferred that we are dealing with “watching TV” activity, whereas having the broom as the object can lead to assume the “sweeping the floor” activity. 

Location is the specific place where an activity occurs, for example, “preparing meals” takes place in the kitchen and “taking a shower” occurs in bathroom. Moreover, the location can help to discard some objects outside the target place. Time is another key characteristic for activity description, since the user may perform an activity in different times but some activities only occur at specific times of the day. For example, the activity “preparing breakfast” usually occurs in the morning, “preparing dinner” usually occurs in the evening and “taking shower” normally occurs once or twice a day: after waking up and/or before going to bed. 

**Figure 3:**
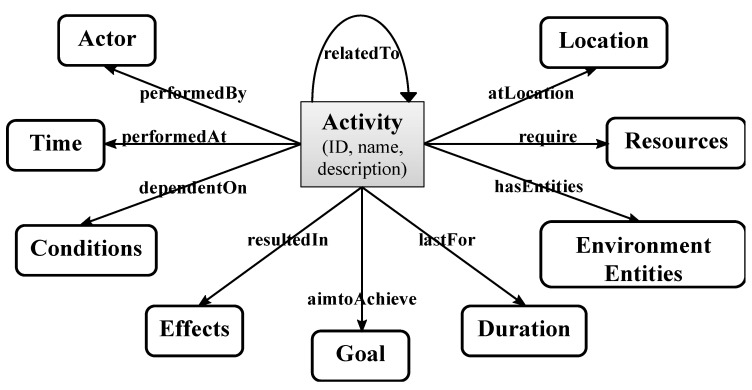
Conceptual description of an activity characterization (based on [69]).

Other context information useful for activities characterization includes human posture, temperature, humidity and speed. Wongpatikaseree *et al.* [68] used “human posture” to help improve the accuracy of the definition of the ADLs in the smart home environment. For example, currently, most of the intelligent activities monitoring systems detect “sleeping” when bed pressure sensors are triggered. However, there is a considerable uncertainty in the “sleeping” recognition defined this way, since the sensors may be triggered by the user while sleeping but also while sitting on the bed. As a consequence, human posture such as “standing”, “sitting” and “lying down” can help to recognize the activity more accurately. Figure 3 (based on [69] and modified by the authors of this paper) depicts the conceptual description of activities characterization in smart home.

Apart from the activity ID, name and textual description, an activity can be described by a number of properties. A property of an activity is specified with its domain and range. This way, the activity can be related to other activities or conceptual entities. Some properties, such as time, object and location, represent the context information within which the activity takes place (note that in Figure 3 “object” is split into physical objects, which are called “Resources”, and alive objects, called “Actors”). Properties like conditions and effects represent the causal and functional relationships, which are used for inference during activity level reasoning.

### 3.3. Activity Context Representation Formalization

The standard formalization of the contextual information defined in the conceptualization process is a key step to allow effective designs and implementations of smart home infrastructures and services. Usually the contextual information related to the activity is not formalized in a standard way due to the heterogeneity of sensor records from different sources. Table 2 shows an example of the differences between three datasets collected from real world smart home environments ([70,71,72]). This heterogeneity hinders interoperability, and the different context representations need separate process approaches to do further reasoning and analysis, which is not efficient for the data exchange and reusability within smart home community. A common context formalization forms the basis for context management to facilitate expressive representation, semantic sharing and interoperability of heterogeneous computational entities. In the following, we present the major methods on activity context representation formalism found in current literature (summarized in Table 3). We will follow, in this sub-section, a bottom-up approach in the sense that we will start by including the most concrete and straightforward structures (key-value pairs) and will end by the most powerful and complex models (ontologies).

**Table 2:** Differences in the format of three datasets collected from real world smart home environments (based on information in [70,71,72]).

References	Sensor ID Coding	Timestamp Coding	Sensor Value Representation	Other Information
Ye *et al.* [70] (based on [71])	Name indicating the location or the object to which the sensor is attached	Date and time in a single feature	ON/OFF (activation or deactivation)	Activity information in the form of annotations (optional)
Cook *et al.* [71]	Number	Date and time in a single feature	ON/OFF (activation or deactivation)	
Tapia *et al.* [72]	Number or name (e.g., “PDA”)	Two timestamps corresponding to the activation and deactivation times. An additional feature shows the difference between them in seconds	Implicit in the activation (OFF to ON) and deactivation (ON to OFF) timestamps	Contextual information (optional): room and object type to which the sensor is attached

The earliest approaches found in the literature on contextual information modelling are the key-value modelling and the markup scheme modelling. *Key-value modelling* is based on the simplest data structure for describing the activity based on flexible units of sensor data representation. A list of simple key-values pairs is used to define the set of attributes and corresponding value mappings as a single record. The key-value modelling approach is adopted frequently in distributed service system architectures (e.g., MAPS [73]), in which the services are described by using a list of attributes. Composite Capability/Preference Profile (CC/PP) [74] is the first W3C standard context modelling approach based on a key-value-based formalism. This specification uses Resource Description Framework (RDF) language and includes elementary constraints and relationships between context types. Particularly, the key-value modelling provides contextual information in a format that is easy to manage. However, it is not expressive enough to capture more sophisticated context data such as relationships, dependencies, time stamping and quality of the context information. Another major limitation is its lack of support for reasoning on context uncertainty or higher context abstractions. 

*Markup scheme modelling* includes a variety of markup languages including XML to define hierarchical data structure, being the hierarchy expressed by means of markup tags with attributes and content. For example, McDonald *et al.* [75] proposed homeML, an XML-based format, as a solution for the heterogeneous nature of data records, in order to support data exchange and storage within smart home environments. They have also developed the homeML suite as an online tool to support data exchange and reuse. Gonçalves *et al.* [76] proposed ecgAWARE, an ECG XML-based markup language, used as an ambulatory electrocardiogram data format standardization to support storage and transmission of ECG data in order to perform patients’ heart telemonitoring during their daily activities. Markup scheme modelling approaches allow one to define interaction patterns and dependencies under the consideration of contextual information, but they suffer from scalability and interoperability problems due to the lack of common design criteria, which are only available at very limited scales.

*Graphical modelling* includes diagrammatical representation of contextual information at design phase. Unified Modelling Language (UML) and Object-Role Modelling (ORM) are commonly used as graphical approaches to appropriately represent activity contextual information by means of a graphical language. Rialle *et al.* [77] used UML context model as the standardization of telemonitoring systems representation to present the components of the system. They developed two types of UML models: the static model, to describe the agents along with their structural relationships, and the dynamic model, to describe the dynamic relationships between agents. The derivation of contextual information from sensor resources, such as profile preferences and location information, is used to identify scenarios. UML models show advantages in capturing information about the static structure and dynamic behaviour of a system, but have limitations in providing precise semantics and supporting reasoning on human behaviours. Henricksen *et al.* [78] developed Context Modelling Language (CML), based on ORM, to allow fact types to be categorized. CML provides graphical notions of different types of contextual information to support the analysis and formal specification of the context requirements, interrelationship representation, grammar support for runtime querying and high-level context abstraction. The CML model also has extended abilities, including capturing different classifications of facts, quality metadata, conflicting assertions (such as conflicting locations from multiple sensors), dependencies among various fact types and histories on the fact types. However, the CML model defines a flat information model in which all the context types are uniformly represented as atomic facts, being as a consequence not appropriate for representing a hierarchical structure. Additionally, it emphasizes the development of context appropriate representations for a particular dominant application, and does not facilitate interoperability with other context-aware systems.

*Object-oriented modelling* uses a set of object oriented programming principles, including abstraction, inheritance, polymorphism, composition, and aggregation, to represent context information. This approach essentially allows to translate between the activity name and the involved objects. For example, the activity “make coffee” is usually related to objects “mug” and “milk”. In object-oriented models entities have related contexts items as attributes or subobjects. An entity is defined as “a person, place or objects that is considered relevant to the interaction between a user and an application, including the user and applications themselves” [79]. Zhang *et al.* [80] adopted a general object-oriented context model to be used in a context-aware smart home system. In their context model, the information is structured around a set of entities, each describing a physical or conceptual object such as a person or an activity. These contextual entities are linked to their attributes and other entities with relationships. For example, a subject (e.g., Ray), that is a contextual entity, may be related to an object (e.g., BedRoom), that is a contextual entity or a datatype value, by means of a verb (e.g., locatedIn), that describes the attributes of the subject or the relationship between the subject and the object. Object-oriented models take advantage of object-oriented features to encapsulate context processing and representation through well-defined interfaces. They also have some drawbacks including the limitation of interoperability and the need of further work in terms of context semantics to be used by services.

*Logic-based modelling* mainly focuses on adding context as logical facts and extracting contextual information by using rules. Bouchard *et al.* [81] proposed a logical framework to represent the contextual information gathered in a smart home using a variety of sensors. Their approach is based on description logic (DL) to overcome the problem of intra-dependencies among context items. DL is used to formalize actions, entities and variables states in smart home environments by creating a hierarchy of contextual information for the specific domain. The logical model consists of a set of conceptual and assertive objects to define the current state of the environment. When new inputs are received from hardware sensors, the agent updates the state of the environment and creates an action structure, representing the changes that happened to the environment. Another logic-based approach is based on event calculus (EC). This approach is a highly developed logical theory of actions that is able to describe dynamically environment state changing in sorted first-order logic. In addition, it has the capability of incorporating a temporal dimension during the description process. Chen *et al.* [82] proposed a formalized framework using EC as the representation approach for the specification of smart home knowledge domain. EC completes the formalization of the domain by using fluents, events, and predicates. Fluents represent any property related to the state of a smart home, events represent any change in a property state and predicates identify the relationships between fluents and events for further formalisation of the domain. In their EC-based framework, they model sensor activations as events and object states as fluents. Additionally they develop a set of high-level logic-based models to represent compound activities, such that the occurrences and effects of compound activities can be formalised in the same way as with primitive actions. This model has clear and elegant semantics in describing contextual information and could serve as a proper way to represent facts, expressions and rules to do further inference and derive new facts from existing action observations. Nevertheless, this model has limitations including its incapability of representing context information uncertainty and its inflexibility to represent multiple users’ activity habits.

*Ontology modelling* provides a semantic contextual information representation of activities. Ontology modelling is particularly interesting to represent activity contextual information since it provides a representation of concepts in a hierarchal manner that has been explicitly agreed upon. Ontology-based markup languages provide a portable specification together with reasoning mechanisms. The core elements in the formalism are concepts, instances and relationships. Standard Ontology for Ubiquitous and Pervasive Application (SOUPA) [83] is one of the most comprehensive ontologies to provide formal and well-structured semantics for context information programming. It contains representation of generic domain knowledge (such as agents, events, space and time) in different scenarios as well as particular concepts in narrower domains (such as home and office environments). Ye *et al.* [84] proposed a reusable formal ontology model to describe the intelligent environment context. The context ontology model consists of four components: object, location, sensor and activity. The structural properties of the activities are represented by the other three concepts (object, location and sensors). Additionally, they specify two types of time conditions: occurring time and duration. For example, the activity “prepare breakfast” should occur in the morning (6 am to 12 am) and the activity “take shower” should not last more than 5 min. Chen *et al.* [85] proposed a formal ADL ontology model to establish links between activities and contextual information through activity-based properties. They used web ontology language (OWL) for ontological modelling and representation. OWL is a formalized markup language theoretically based on the well-developed knowledge representation formalism of DL. It is primarily designed to represent contextual information about classes of objects and inter-relationships between them. The main feature of their ontology model is that it can model domain knowledge at two levels of abstraction: the conceptual level, in which an activity class is described by a number of properties according to generic activity knowledge; and the specific level, in which the special way a user performs an activity can be modelled as an instance. A similar work can be found in [65]. Ontology-based representations approaches show clear advantages for context knowledge sharing among different entities by using OWL formalisms. They also work well as a solution for capturing sensor information in terms of heterogeneity, interoperability and usability with user-friendly graphic tools (e.g., protégé [86]). However, there are current limitations of this domain ontology models that must be tackled: they require solid knowledge engineering skills; they do not support time-related reasoning, they are normally computationally expensive in context reasoning; they have limited ability to deal with uncertain and changing context of objects.

**Table 3:** Context representation: options for its formalization.

Type	References	Advantages	Disadvantages
Key-value modelling	Aiello *et al.* [73] CC/PP [74]	Key-value pairs are easy to manage	Limited capacities in capturing sophisticated context types
Markup scheme modelling	McDonald *et al.* [75] Gonçalves *et al.* [76]	Allow defining interaction patterns	Lack of design criteria, only available in a limited scale
Graphical modelling	Rialle *et al.* [77] Henricksen *et al.* [78]	More comprehensive support for capturing imperfect and historical information	Flat information model, limited in supporting interoperability within context-aware systems
Object-oriented modelling	Zhang *et al.* [80]	Good performance in object related activity context representation	Limitation of interoperability
Logic-based modelling	Bruno *et al.* [81] Chen *et al.* [82]	Clear and elegant semantics in describing contextual information	Unable to represent uncertain context and inflexibility to represent user’s habits
Ontology-based modelling	Okeyo *et al.* [65] Ye *et al.* [84] Perich *et al.* [83] Chen *et al.* [85]	Represent context in terms of heterogeneity, interoperability, and usability with user friendly interface	Require well-built knowledge engineering skills, limited ability in dealing with uncertain and changing context

## 4. Sensors in the Smart Home

The concept of ubiquitous sensing arises in the smart home, in which a wide variety of sensors/devices integrated in daily objects and infrastructure at home is connected by network technologies in order to gather contextual information about human activities. There are mainly two categories of activity monitoring approaches in terms of sensor deployment: Audio/visual-based approach and sensor-based approach. The audio/visual-based approach involves audio and visual sensing devices, such as microphones and cameras, to monitor the inhabitants’ movements and environmental changes. On its side, the sensor-based approach is based on the use of sensors embedded in the smart home environment or worn by the users. Environmental sensors are used to detect human activities related to specific objects or performed in specific areas, whereas wearable sensors are used for monitoring ambulatory activities and physiological signals. In this section, we will summarize the commonly used sensors for monitoring different activities in recent research and analyse the advantages/disadvantages of these sensors. The main characteristics of these sensors are summarized in Table 4.

**Table 4:** Sensors in the smart home: summary of main characteristics relevant to activity detection.

Sensor	Measurement	Data Format	Advantages	Disadvantages
Video cameras	Human actions/environmental state	Image, video	Precise information	Privacy issues, computational expense, acceptability issues
Microphones	Voice detection, other sounds	Audio	Certain and rich information about sound	Implementation difficulty and high computation cost, potential acceptability issues
Simple binary sensors	User-object interaction detection/movements and location identification	Categorical	Low-cost, low-maintenance, easy to install and replace, inexpensive, less privacy-sensitivity, minimal computation requirements	Provide simple and limited information for composite and multi-user activity monitoring
RFID	Object and user identification	Categorical	Small size and low cost	Reader collision and tag collision, range limited
Temperature sensors/light sensors/humidity sensors	Environmental parameters	Time series	Intuitive monitoring of environment and object	Limited information for activity monitoring
Wearable inertial sensors	Acceleration/orientation	Time series	Compact size, low cost, non-intrusiveness, high accuracy, unique identification of users, user’s location easily tracked.	Cumbersome and uncomfortable feeling, cannot provide sufficient context information
Wearable vital signs sensors	Vital signs	Analog signal	Sensitive to slight change in vital signs monitoring, more accurate in emergency situation detecting	Reliability constraints, Security issues and uncomfortable feeling for long-time skin attaching

Video cameras are low-cost devices that can provide very detailed and rich context information about human actions and environmental states. A sequence of images is directly used to detect human activity within their monitored areas. Video information can provide direct and clear information about the objects within smart home, for example, the number of people. Thus video cameras have strong advantages on multiple-users’ activity monitoring. However, they face difficulties including privacy issues, high computational expense and environment dependency. On their part, microphones have as advantages their ability of providing accurate information about users’ communications and sounds in specific locations inside smart home. However, they suffer from implementation difficulties and high computational costs associated to the audio processing algorithms necessary to distinguish different sounds, especially when there are multiple residents inside the home. Microphones, although probably to a lesser extent than video cameras, can also be perceived as privacy threats, since they can potentially record private conversations. It is anyway worth mentioning that there are studies that show that the acceptability of this technology is strongly influenced by the increase in freedom that caregivers’ perceive while preserving safety of their loved elderly [87] and the potential usefulness of voice-driven interfaces inside home [88].

We firmly believe that the acceptance of the technology by the users is a critical issue in smart home environments (as already stated in Section 2.4 above). That is the reason why our analysis is mainly focused on sensors other than the audio/visual ones. 

### 4.1. Environmental Sensors

Diverse sensors can be deployed in different home areas or attached on a range of objects to monitor activities in smart home. Most ADLs are performed in specific locations and with specific user-object interactions. For example, cooking activity usually takes place in the kitchen (specific space), and telephoning involves interacting with the phone (specific interaction with an object). Thus the activity can be recognized from user-object interactions combined with environment observation. For example, if sensors indicate that the stove is on and that there is water usage in the kitchen, it can be strongly suggested that the activity of preparing meals is taking place. Therefore, it is assumed that environmental sensors data can constitute powerful information to observe the human behaviours within smart home. In this section, we will summarize the commonly used sensors embedded in smart home for detecting ADLs and discuss their advantages and disadvantages.

Simple binary sensors, including state-change sensors, motion sensors, contact switches and pressure sensors, may be deployed on a range of objects in smart home environments for monitoring users’ movements and locations. A simple state-change sensor can be used to detect any change of the state of an object which can be subsequently be used to reflect the user-object interactions. For example, a state-change sensor attached to the handset of a telephone detects if the handset has been lifted from the telephone base station. Motion sensors are used for detecting the inhabitant’s presence and location throughout the house. Infrared presence sensor is the most commonly used type of motion sensor in smart environments to detect users’ presence. Contact switches are usually installed on the doors of rooms, fridge and cabinets for detecting specific interactions that the user performs with these objects. Pressure sensors can be discreetly installed on objects such as sofas, beds, chairs and floors for tracking movements and locations of the user. For example, the usage of pressure sensors on the floor in front of the kitchen sink could serve for detecting the meal preparation activity with the help of other sensors. 

In real-world scenarios using a single sensor type normally cannot provide enough information for detecting activities, especially for some complex ones. Thus multiple sensors are needed to provide more accurate information related to activity monitoring. Wilson and Atkeson [89] chose four kinds of binary sensors (motion detectors, break-beam sensors, pressure sensors and contact switches) which can be triggered by gross movement, point movement, gross manipulation and point manipulation for tracking and monitoring activities. Ordónez *et al.* [90] monitored seven daily activities (leaving house, using toileting, taking shower, sleeping, eating breakfast, having dinner, and drinking) by using three kinds of sensors. Passive infrared sensors are used to detect motion in a specific area, reed switches are used to detect open/close states of doors and cupboards, and float sensors are used to measure the toilet being flushed. These low-cost, easy-to-install and long-lived binary sensors exhibit the advantages of unobtrusive user-object interaction monitoring in a privacy-preserving way. In addition, they are easy to replace and the gathered data require minimal computation resources. The main drawback is that they can only provide very limited information especially for composite and multi-user activity monitoring.

Radio-Frequency Identification (RFID) works as a combination of environmental sensor and wearable sensor technologies. It consists of a reader worn by the user and an electronic tag attached to an object. The tag responds to a unique identifier, electronically stored in memory, when interrogated by a reader. In smart home both passive and active RFID tags may be used. A passive RFID tag does not contain a power source and is usually attached to an object for detecting the interaction between a user and the object. An active RFID tag contains a battery and is often carried by a user for personal identification throughout the house. Fujinami *et al.* [91] inserted RFID tags into slippers to track the long-term daily life activities of the elderly with dementia in a group home in Japan. Philipose *et al.* [92] developed a system to monitor activities in a home environment by using RFID tags attached to the objects. In this system, the activity information is presented with a probabilistic sequence of the used objects. Kim *et al.* [93] proposed an indoor ubiquitous-healthcare system (U-healthcare) based on RFID technology to accurately locate and monitor in real-time the elderly’s whereabouts. They analyse the locations in association with the time slots and the length of time the user stays in the same place. Then, the collected data is used to infer information such as movements and activity patterns, and determine the elderly’s wellbeing. RFID has as obvious advantages its small size (it can be placed out of sight) and low cost. Moreover, due to the unique identification, RFID is well suited for tracking multiple-users’ location and interactions in order to support activities monitoring of several people [94]. RFID also has disadvantages, such as reader collision and tag collision. Reader collision is produced when the tag is being read by several RFID readers simultaneously, being unable to answer to them. Tag collision usually occurs when a large volume of tags are read by the RFID tag reader and this cannot distinguish these signals. In addition, the problem of reliability and stability, especially when reading through liquid and metals, is another limitation of RFID.

A variety of other sensors such as light sensors, temperature sensors, humidity sensors or power sensors have been also deployed and used in smart home environments to help in the detection of activities. Light sensors are used to measure the intensity of light in a specific location. Temperature sensors are used to measure the temperature of the object and its surrounding environment. Humidity sensors are used to detect the air humidity in a specific area. Power sensors are used to detect the usage of electric devices. These sensors can perform intuitive monitoring of environment and object, but on their own they only can provide very limited information for activity monitoring.

### 4.2. Wearable Sensors

Wearable sensors refer to sensors that are attached to human body either directly or indirectly, and which usually provide a continuous flow of information. Their small size allows that they are embedded into belts, clothes, glasses, wristwatches, shoes and mobile devices to make them easier to wear. These sensors can be divided into inertial sensors and vital sign sensors (or biosensors). Wearable inertial sensors can give accurate descriptive features of user’s movement and body posture. Vital signs collected from wearable biosensors such as heart rate, blood pressure and skin temperature are critical for elderly people’s health condition monitoring. In this part, we summarize the most commonly used wearable sensors for monitoring ambulatory activities and vital signs.

#### 4.2.1. Inertial Sensors

Accelerometers are the most frequently used sensors for ambulatory activity monitoring. They can measure the value of acceleration along a sensitive axis and are particularly effective in monitoring activities related to body motion such as doing exercise, walking, standing, sitting, or walking upstairs and downstairs. Data collected from accelerometers has four attributes: time, acceleration from x-axis, acceleration from y-axis and acceleration from z-axis. They can provide information to indicate human movement responding to frequency and tilt, which is critical to assess the posture. Due to their small size and relatively low cost, accelerometers can be embedded into wrist bands, watches, bracelets and belts to monitor the user’s activities and wirelessly send data to mobile computing devices. The context inferred from these data can be used for long-time continuous activities monitoring and emergency situation recognition such as fall detection. Recent researches make a try to place accelerometers on different body parts for identifying the optimal activity monitoring performance. For example, in [95], Zhu and Sheng monitored eight daily activities (sitting, standing, lying, walking, sit-to-stand, stand-to-sit, lie-to-sit, and sit-to-lie) in an indoor apartment environment. They use an accelerometer worn on the right thigh of the user to collect motion data. Mannini and Rosenberger [96] used a single accelerometer placed at the wrist or ankle of the user to collect raw data for long-time monitoring. Lei and Bourke [97] placed four accelerometers on four different parts of the body (chest, left under-arm, waist and thigh) to monitor and recognize five activities (standing, sitting, lying, walking and transition. Additional recent research and experiments about different placements of accelerometers are detailed in Table 5. 

Gyroscopes use a small vibrating mass inserted into the sensor for measuring angular velocity and maintain orientation. The change of the angle compared to the initial known value can be detected over a period of time. Gyroscopes have some limitations including output drift over time, output offsets when the device is in static state, as well as the limitation of sensitivity to a particular range of angular velocities. In the field of ambulatory activities monitoring, most researches are focusing on the usage of accelerometer sensors or the combination of accelerometer and gyroscope sensors. Narayanan *et al.* [98] used a triaxial accelerometer attached to the waist to measure timed up-and-go test, sit-to-stand with five repetitions and alternate step test with the aim of estimating the fall risk of the elderly. Greene [99] utilize accelerometers and gyroscopes attached to the user’s two legs to measure the timed up-and-go test in order to distinguish fallers from non-fallers. Zijlstra *et al.* [100] used accelerometers and gyroscopes to determine vertical accelerations and power of sit-to-stand movements while rising from a chair. This approach is relevant to fall detection and mobility assessment of elderly people, and a similar work can be found in [101]. Varkey *et al.* [102] detected six different activities by placing accelerometers and gyroscopes on the right arm wrist and right foot of the user to collect linear and angular accelerations.

**Table 5:** Different placements of accelerometers on human body for activity monitoring.

References	Number of Accelerometers	Placements	Activities
Gjoreski *et al.* [103] (2011)	7	Chest, left thigh, right ankle	Standing, sitting, lying, going down, standing up, sitting on the ground, on all fours
Jiang *et al.* [104] (2011)	4	Left forearm, right forearm, left shank and right shank	Standing straight, sitting on a chair, lying on a bed, walking, jogging, cycling, walking on an elliptical machine, running on an elliptical machine, rowing and weight lifting.
Jennifer *et al.* [105] (2011)	1	Smartphone	Walking, jogging, upstairs, downstairs, standing, sitting
Zhu and Sheng [95] (2011)	1	Right thigh	Sitting, standing, lying, walking, sit-to-stand, stand-to-sit, lie-to-sit, sit-to-lie
Siirtola *et al.* [106] (2012)	1	Smartphone placed in trousers’ front pocket	Walking, cycling, sitting, standing, driving a car
Hemalatha and Vaidehi [107] (2013)	1	Chest	Standing, walking, sitting, lying, fall
Mannini *et al.* [108] (2013)	1	Wrist/ankle	26 daily activities
Zheng *et al.* [96] (2013)	1	Wrist/hip/waist pocket	Lying, sitting, standing, walking, running, dancing, jogging, upstairs, downstairs, skipping
Muaaz *et al.* [109] (2014)	1	Waist, right-hand side of the hip	Walking
Gao *et al.* [97] (2014)	4	Chest, left under-arm, waist and thigh	Lying, sitting, standing, flat walking and up & down stairs, lie-to-stand, stand-to-lie, sit-to-stand, stand-to-sit

In terms of activity monitoring, the system benefits from some features of inertial sensors including their compact size, low power requirements, low cost, non-intrusiveness and the capacity to provide data directly related to the motion of the user. However, inertial sensors also suffer from limitations. The placement of inertial sensors on diverse positions may result in cumbersome and uncomfortable feeling, which may lead to low acceptance by the older adults. Most wearable inertial sensors need to collect data continuously, thus the battery life and effectiveness of the device may become a great challenge. Using inertial sensors cannot in many occasions provide sufficient context information, especially when monitoring complex motions and activities that involve multiple interactions with environment objects.

#### 4.2.2. Vital Signs Sensors

Monitoring activities through vital signs provided by biosensors is recently gaining research interest. In smart homes, some vital signs such as Electrocardiogram (ECG), heart rate, blood pressure, blood glucose, oxygen saturation and respiratory rate are related to healthcare services. There are various biosensors used to measure the wide range of vital signals: Electroencephalography (EEG) sensors for monitoring electrical brain activity, Electrooculography (EOG) sensors for monitoring eye movement of ocular activity, Electromyography (EMG) sensors for monitoring muscle activity. Electrocardiography (ECG) sensors for monitoring cardiac activity, pressure sensors for monitoring blood pressure, CO_2_ gas sensors for monitoring respiration, thermal sensors for monitoring body temperature and Galvanic Skin Response (GSR) for monitoring skin sweating. These vital sign parameters can help monitor the user’s health status during the execution of activities [110]. For example, regarding the brain electrical activity monitoring, delta, alpha and beta waves are mainly used to detect sleep state, panic disorder, and sudden unexplained nocturnal death syndrome. Ocular activity gives information on the state of the user by detecting eye movements: rapid eye movements would indicate that the person is awake, and slowly rolling eye movements would indicate that the user is in the state of transition from being awake to sleeping. Muscle activity may occur in different parts of the body. For instance, a more intense activity of chin muscles may represent swallowing or drinking. Cardiac activity mainly focuses on heart rate monitoring, which gives indications of arrhythmias, and blood pressure monitoring, which shows immediate changes of user’s health state such as nose bleeding. Respiration monitoring involves measuring airflow though nose and mouth. Skin temperature is a typical way of detecting fever and skin sweating is a good indicator of sport and housework activities.

Matthews *et al.* [111] designed a wearable physiological sensor suite (PSS) to monitor long-term state for patients with cardiac or neurological conditions. This PSS measures four bioelectric signals: ECG, EEG, EOG and EMG. Heart rate derived from ECG sensor (integrated into a belt) is used as an indicator of health-threatening values, EOG sensor (integrated into a glass) is used to monitor eye blinking and EMG sensor is placed on the subject’s bicep to monitor muscle activity. Tartarisco *et al.* [64] use ECG sensor embedded into a wearable device to provide electrocardiographic signals for the evaluation of the stress state of the user during the execution of daily activities. Based on the data collected from these biosensors, services such as further disease prediction, anomaly detection and diagnosis decision-making can be provided.

Biosensors, like other wearable sensors, have as advantages their low cost, low error levels, non-intrusiveness and high accuracy. Besides, they are very sensitive to slight changes of physiological signals, and thus they can support non-invasive alternatives for continuous healthcare monitoring in smart home environments. The disadvantages of biosensors include reliability constrains and uncomfortable feeling for long time skin attaching.

## 5. Sensor Data Processing

Raw data obtained from sensors has to be processed in order for it to be of any use during the later phases (context identification, activity modelling, and learning/reasoning phase). Moreover, this processing has critical impact on output accuracy and recognition results of the whole process, being thus crucial for the success of the complete system.

Current research on SH focuses primarily on learning algorithms and reasoning approaches. There are not so many papers devoted to the processing actions which sit between raw sensor data and activity recognition. Furthermore, we think that it would be highly beneficial to advance towards a formalization of what processing algorithms are more adequate depending on the concrete sensors and activities to be detected in SH scenarios. 

**Figure 4:**
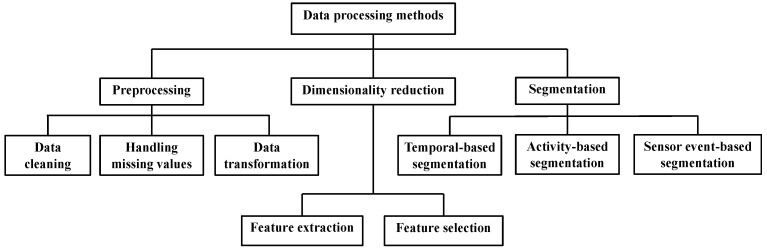
Sensor data processing methods classification.

The most common processing methods are related to proper data preprocessing, segmentation and dimensionality reduction methods (as shown in Figure 4). In this section, we will analyse these methods, reviewing current literature and extracting the significant information to pave the way for the abovementioned formalization. We finalize Section 5 with a summary table regarding this objective.

### 5.1. Data Preprocessing

Data collected from various sensors is inherently noisy. Data preprocessing operations mainly include data cleaning to remove the artifacts and unwanted samples, data interpolation to cope with missing readings and data transformation to put data into the proper format.

#### 5.1.1. Data Cleaning

Raw data gathered from sensors has a certain degree of erroneous, noisy and redundant information caused by discharged batteries, failures in sensor readings and intermittent communication loss in wireless sensor networks. To compensate for these effects, data cleaning is necessary. This set of techniques is used to smooth out raw data by filtering out artifacts and removing unwanted information, in order to only keep the characteristics of signals that carry relevant information. To do so, several filters are used which have to be in accordance with the characteristics of the sensor. We provide in the following a description of the main techniques used for data cleaning, together with their relationship with the type of information gathered and the objective of it.

Regarding scenarios with presence sensors, Wilson and Atkeson [89] used both Bayes and particle filters to preprocess the data from four binary sensors and a RFID sensor. The results show that the Bayes filter works well on tracking a single user in a noisy environment whereas the particle filter works better on scenarios with multiple users. Noury and Hadidi [112] used a median filter to remove nonlinear artifacts. They also use a “first order hold” (FOH) filter to remove redundancy in the dataset collected from presence sensors placed in different areas in a smart home. Guettari *et al.* [113] also used a median filter to avoid abnormal measurements of passive presence sensors used to detect human presence within a smart home. 

The 3D acceleration data collected from any accelerometer contains a constant component due to gravity. The effect of gravity should be eliminated in order to obtain the real acceleration experimented by the device. Khan *et al.* [114] applied a low-pass filter to isolate the constant gravity acceleration in the time series dataset collected from accelerometers. Wang *et al.* [115] compared the noise reduction performance of four filters (median filter, Kalman filter, low-pass filter and discrete wavelet package shrinkage) on the acceleration signals captured by a nine-axis wireless body sensor network platform. The results show that, in terms of signal-to-noise ratio (SNR) and correlation coefficient (R) between filtered and reference signals, Kalman filter exhibits the greatest figures, followed by the median filter and the discrete wavelet package shrinkage, the low-pass filter has the worst performance due to the waveform delay it causes. The authors also found that window length has great impact on the real-time performance of the median filter and the inappropriate choose of filter order and cut-off frequency would lead to large waveform delay of low-pass filters.

ECG signals usually contain unwanted interference such as electrode noise, muscle artifacts and line noise. Removing these unwanted interferences is helpful for accurate diagnosis and decision making. Sen and Chandrakar [116] used an extended Kalman filter to remove noise from ECG signals. When compared to median filter, the extended Kalman filter provides very low mean square error and very high peak signal to noise ratio. In [117], since EOG signal information corresponding to basic eye movements lies in the frequency range 0.5 Hz–35 Hz, the authors use a high-pass filter with cut-off frequency = 0.5 Hz and a low-pass filter with cut-off frequency = 35 Hz to keep the interesting frequency band. Bulling *et al.* [118] compared the performance of median filter, low-pass filter and wavelet package shrinkage on real-time EOG signals. They conclude that the median filter provides the best results without introducing any artificial signal changes. The selection of window size is again found to be critical. It should be small enough to retain short signal pulses. Khushaba *et al.* [119] applied a bandpass filter between 20 and 450 Hz on EMG signals to remove noise. In addition, a notch filter is used to remove the 50 Hz power line interference.

There are two important issues related to datasets obtained from smart environments which may highly impact on further data analysis and classification accuracy. These problems are called class imbalance (*i.e.*, classes which exhibit much more samples than others, causing uncompensation in training) and class overlapping (*i.e.*, a subset of the samples may be associated to several different classes with similar probabilities, causing ambiguity) [99]. For overcoming this issue, Barnan *et al.* [120] proposed a novel approach, named clustering-based under-sampling (ClusBUS), which they apply to the CASAS smart home dataset to deal with the overlapping data points in the presence of imbalance class distribution. Compared to conventional methods which interpret boundary points as noise, the ClusBUS adopts an under-sampling method based on clustering, able to identify “interesting” clusters in the instance space and ambiguous data regions where samples in the minority are embedded into the majority class. The results show that ClusBUS can provide more importance to the minority class by discarding majority samples from the ambiguous data regions. Goodwin *et al.* [121] also used under-sampling technique to deal with the unknown classes in an acceleration dataset. In their experiment the majority class consists of not labelled motor movement samples, and under-sampling is performed on them to balance the dataset by discarding a proportion of the samples in this class.

#### 5.1.2. Handling Missing Values

Data collected from sensor networks sometimes has missing values. For instance, almost 30% of the readings are lost in typical RFID datasets. These missing readings can be interpolated with proper values. Linear interpolation is the most commonly used technique to fill in the missing values. Parlak and Marsic [122] used passive RFID tags to detect object motions for trauma resuscitation, where they face the problem of irregular intervals tag readings produced by irregularities in data arrival and signal transmission delay. The authors use linear interpolation to fill in the missing samples within each window, being the number of interpolation points determined by the window size. Muaaz and Mayrhofer [109] used a Personal Mobile Device (PMD) to measure parameters related to users’ walking. The raw data is only collected when Android API is triggered, and thus the accelerometer sensor does not provide data at equal intervals. They adopt linear interpolation techniques to reshape acceleration data into equal intervals, and this reshaped data can also be oversampled to avoid relevant values loss.

Other interpolation techniques include nearest neighbour interpolation and cubic interpolation. For instance, Shayei *et al.* [123] proposed to apply cubic spline interpolation on R-waves present in the ECG signals to derive the respiratory signal. Their results show that the algorithm both reduces the computational complexity and preserves the same performance level.

#### 5.1.3. Data Transformation

In order to perform further data processing and analysis, data has to be put into the proper format. For example, the values obtained directly from a temperature sensor are in numeric format, which cannot be used in some data mining algorithms. Some of these algorithms need more suitable types of attributes such as nominal or categorical values. The function of some of the data transformation methods is precisely to convert raw data into appropriate attributes format in accordance to the system requirements. Rodner and litz [124] used association rule mining approaches to model typical behaviours of inhabitants for ambient assisted living systems. The data collected from a motion detector with integrated lux meter has a format (timestamp [numeric], motion [binominal] and lux [numeric]) containing numeric data which cannot be used in rule mining. The authors convert observed numeric values into nominal attributes transforming, for example, the numeric lux values into categories such as “bright” or “dark”. As another example, Sun and Zhang [125] transformed the raw analog signals collected from ECG to digital attributes for further signal analysis.

Another commonly used data format transformation approach is data normalization, which is required for obtaining a representation format of sensor output values that allows their quantitative or qualitative direct comparison. Due to the enormous range of different possible scales, data normalization is performed considering the following criteria [126]:

1. Nature of scale, which refers to the basic mathematical properties of the used scales.
2. Homogeneity, which refers to whether, or not, the sensors are measuring the same physical phenomenon.
3. Empirical statistical distribution observed in the sensor values when applied to a given population segment (e.g., the elderly).
4. Semantics, which refers to how the scale should be interpreted, such as probabilistic, possibilistic, utility or degree of similarity.

### 5.2. Data Segmentation

Data from sensors usually comes as a continuous flow of raw data. This is because sensors will basically provide instant measurements of the monitored phenomenon, either when requested or at periodic intervals. One of the main challenges related to sensory data preprocessing is to achieve a proper division of this raw and continuous data flow into smaller blocks of information. This is the purpose of segmentation methods. The proper selection and parameterization of segmentation techniques has great potential impact on the success of feature extraction and inference algorithms, directly resulting in the accuracy of activity monitoring and recognition. Current literature highlights mainly three categories of segmentation approaches: temporal-based segmentation, activity-based segmentation and sensor event-based segmentation, as explained in the following sections.

#### 5.2.1. Temporal-Based Segmentation

There are two main temporal-based segmentation approaches: time interval-based segmentation and sliding window segmentation. Time interval-based approach consists in dividing the sensorial datasets into chunks of equal time duration. This approach is commonly used, for example, for breaking down the temporal stream data obtained by accelerometer and gyroscope sensors. The activities monitored with these sensors are related to human actions but the stream dataset provided by them has an important temporal component that must be properly managed. Ordóñez *et al.* [127] used 60 s as the time interval to segment the dataset from the binary sensors embedded in a smart home. This interval length is chosen by taking into account activity discrimination and accurate labelling criteria. Noury and Hadidi [112] segmented the raw data into days to create a daily matrix from the monthly dataset. Then they go for a six-hour interval to divide the daily matrix into four time slices for further data processing and analysis. Krishnan and Panchanathan [128] divided the acceleration data stream into frames of 5.12 s’ duration.

In general the time interval-based windowing approach is appropriate for data streams that are continuously obtained by sensors over a relatively long period of time, and it has the advantages of less computation complexity and cost. However, the selection of the optimal value for the time interval is very critical. A too small interval may split one activity into two adjacent windows, especially for a long duration activity such as cooking, resulting in a lack of sufficient information to arrive to an accurate classification. On the other hand, a too wide interval may gather two or more activities into the same segment and add extra noise for further data processing. Another difficult issue of this approach is the development of effective heuristics for deciding when an activity extends over two intervals, especially when sensors do not have a constant sampling rate.

The sliding window approach is one of the most widely used segmentation techniques for activity monitoring. By using this approach, the continuous sensor data flow is divided into windows with either static or dynamic sizes. In the former case, the fixed window size (used to retrieve sensor features) can be based on either equal time intervals or equal number of sensor events. Three segmentation algorithms are of particular interest in current literature: Fixed-size Non-overlapping Sliding Window (FNSW), Fixed-size Overlapping Sliding Window (FOSW) and Sliding Window And Bottom-up (SWAB) [129]. FNSW is a simple segmentation approach without any data overlap, thus the amount of windows can be exactly calculated (if the complete time interval or the complete number of samples is known). Van *et al.* [130] used FNSW to divide the stream dataset collected from a smart home environment, the results showing good performance for recognizing the activities “leaving home”, “toileting”, “showering”, “sleeping”, “having breakfast”, “having dinner” and “drinking”. However since this algorithm works with a fixed window size, the data associated with a particular event such as a fall could be split in different windows, resulting in important loss of information. FOSW is an improvement of FNSW which introduces flexibility to solve some of its main limitations. FOSW includes data overlap between adjacent windows, and the different overlapping percentages can be referred to as window shifts. These values may vary from 0% to 100%. FOSW with 0% overlapping is equal to the FNSW segmentation technique, whereas a 100% overlap is an extreme value with limited usefulness since in this case windows would not slide at all, being the same data always included in all of them. Note that FOSW with 0% overlap and fixed time-based size actually boils down to the time-interval based segmentation approach. SWAB segmentation tries to combine the speed of the aforementioned simpler sliding window algorithms with the more accurate segmentation that would stem from a complete offline processing of the data stream. It does so by working with a buffer of samples that normally is wide enough to contain several segments. This buffer is emptied “from the left” and refilled “to the right” with a number of samples (or a time interval) that depends on the structure of the data.

Achumba *et al.* [131] set up an experiment to compare the classification accuracy of FOSW with different window overlap values (0%, 25%, 50%, 75% and 90%) applied to acceleration data for recognizing ambulatory activities. Their work determine that the optimal segmentation approach is FOSW with 90% of window overlap and 12 s of window size, reaching a classification accuracy of 98.38%. They also found that SWAB segmentation scales linearly with the amount of data and produces high quality approximations at the expense of more complex implementations (compared to FOSW and FNSW). 

Dynamic sliding window methods enable varying window sizes based on different sensor features (such as sensor state change or location change of consecutive sensor data) and/or on typical activity duration. Laguna *et al.* [132] proposed a dynamic sliding window approach by using significant events to define the window boundaries. This approach adjusts dynamically not only the window sizes but also the shift to apply at every step. Krishnan *et al.* [133] proposed and evaluated a combination of static and dynamic sliding window sizes. They improve the sliding window approach with three modifications that capture the relationship between sensor events within a window and between multiple windows. The three modifications are oriented to calculate the size of the window to be used at each moment depending on the sensor data, the environment and the monitored activity. In order to achieve this, they consider the following information for the selection of the window size: time-based weighting, mutual information-based weighting, activity probabilities in the previous window and previously detected activity. The results on several real-world smart home datasets suggest that the adding of past contextual information to the process leads to enhanced performance for streaming activity recognition. 

The sliding window segmentation approach is especially beneficial when dealing with activities with an important periodic component (such as walking and running) and also in the case of static activities (e.g., sitting and standing). The computational complexity of the reasoning performed on segmented data depends on the number of samples within each window, thus there is usually a tradeoff between performance and accuracy of activity monitoring systems that use segmentation methods. In current literature, a wide range of window sizes are adopted depending on the different monitored activities. [105] uses sliding window segmentation with window size = 10 s to divide the dataset collected from an accelerometer embedded into a smartphone. The results show an efficient accuracy on recognizing activities such as walking, jogging, standing, sitting, walking upstairs and downstairs. The same window size is used in [96]. In [106], a window size of 7 s is used on acceleration dataset to monitor ambulatory activities such as walking, cycling, sitting and standing. Authors of [104] use a windows size of 6 s for dataset collected from four accelerometers placed in different parts of the body. The smallest window sizes, such as 4 s [108], 3.88 s [37,63], 2 s [108], 1.5 s [134], 1 s [97,103,135] and 0.08 s [136] are also used to segment acceleration datasets. Intuitively, decreasing the window size allows for a faster segmentation process and activity detection, but it would trigger the execution of learning algorithms more frequently resulting in higher overhead. Banos *et al.* [137] evaluate different window sizes used for activity recognition with a non-overlapping sliding windowing approach. These sizes range from 0.25 to 7 s in 0.25 s steps, and the concrete interval at each moment is chosen according to the values used in previous activity recognition systems. As a result they proved that short windows normally result in better recognition performance, and the 1 to 2 s interval exhibits the best trade-off between recognition speed and accuracy from a global perspective. Generally, from previous work we may infer that activities with high motion variability (e.g., household activities) normally require longer window sizes whereas activities related to the observation of specific body parts (or the whole body) (e.g., walking, running, cycling) benefit from shorter window sizes. As a final remark on these techniques, it is significant to note that temporal-based segmentation works well on single user sequential datasets, but it has limited ability on segmenting multi-user datasets containing concurrent and interleaved activities.

#### 5.2.2. Activity-Based Segmentation

Activity-based segmentation consists in dividing the sensory data stream by identifying the start and end points of each activity. As a consequence, the main issue to be tackled for these methods is the correct identification of those boundary instants. Various methods are proposed in the literature to identify the beginning and ending points of activities. Yoshizawa *et al.* [138] proposed a method to identify these limits, distinguishing between static activities (such as standing and sitting) and movement-related ones (such as walking and running). In their approach, a threshold is set for detecting the changing points of static activities, and the analysis of variations in the frequency domain is used for determining the beginning and ending points of movement-related activities. Similar work can be found in [139], where the authors propose a model based on wavelet decomposition to separate into segments the continuous data records collected from an accelerometer. This model detects frequency changes for the location of three walking activities’ limits (walking, ascending stairs and descending stairs). Another method to identify the beginning and ending points of an activity is achieved by asking the user to give explicit feedback. In [140], volunteers in the experiments are asked to stand still for a few seconds between two activities to identify these transition points. A similar method is used in [141], where the subjects are asked to set the beginning and ending points of daily life activities such as cooking and cleaning though a smart phone interface. Obviously, this method is only feasible in laboratory environments. Moreover, the participants perform the activities following instructions from experts, thus leading to potentially artificial behaviors (*i.e.*, not totally equal to the ones that would be observed in real life).

#### 5.2.3. Sensor Event-Based Segmentation

Sensor event-based segmentation approaches are used for recognizing activities which consist of a sequence of movements, events or actions that take place in a certain time order and that might be interleaved with other activities’ events, for example, “household” or “meal preparation” activities. Compared to temporal-based segmentation, in this case the events that form the activity may not be distributed uniformly in time and may occur sporadically, thus the size of the windows is not fixed. For sensors embedded in smart environments, Fahim *et al.* [142] divided the data streams into sequence windows with equal number of sensor events, being the duration of each window dependent on the duration of the individual events. Their approach considers the possibility that there may be no sensor readings triggered in a period of time during the execution of activities, but it treats all the sensor events with equal importance. To overcome that problem, Tapia *et al.* [72] proposed a method to explicitly incorporate the activity time span calculated as the average duration taking into account all the activity labels generated by Experience Sampling Method (ESM) and indirect observation. Hong and Nugent [143] developed three algorithms to segment time series events by extracting into each segment the consecutive sensor events associated with a complete activity. Their first proposal is based on the location perspective of activities, by assuming that “if two conjunctive sensor events are part of an activity, the locations indicated by two sensors should both belong to the set of locations that the activity can be performed in, otherwise the sensors contribute to the different activities”. The second segmentation approach they describe is model-based, and is based on modelling each activity in terms of sensor activation to determine if two consecutive sensor readings are involved in the same set of activities. Their third proposed algorithm is based on the predominant sensor associated with an activity, being the predominant sensor the most important one during the activity time span.

In order to segment concurrent datasets, segmentation approaches based on similarity measurements between sensor events have obtained more research attention. Ye *et al.* [84] developed a semantic-driven online sensor data segmentation method based on temporal, spatial and object semantics of sensor events. These semantics are used to evaluate the semantic similarity between two adjacent sensor events and to consequently divide, in a dynamic manner, a sequence of raw sensor events into segments. Each segment will then be mapped to one activity. Stevenson *et al.* [70] proposed a real-time multi-user segmentation mechanism based on sensor and activity semantics to partition a continuous sensor sequence into fragments. In this case the segmentation is done by using event patterns obtained from the semantic dissimilarity among sensor events. Temporal knowledge described in the generic ontological models is used to evaluate the semantic similarity between consecutive sensor events and control the time interval of fragments. Compared to the static sliding window approaches, their segmentation approach can detect the boundary of concurrent activities more accurately, as well as produce a smaller number of partitions better adjusted to real activities.

### 5.3. Dimensionality Reduction

Dimensionality reduction operations include the extraction of those features that represent the significant data characteristics and the posterior selection of specific discriminative features to reduce the dimensionality of the feature vector while maintaining most of the relevant information. The rationale behind these methods is related to the huge volume of raw data that may be generated in smart homes, due to the heterogeneity and ubiquity of sensors used. The ability of extracting summarized and useful information from raw sensor data becomes a key factor for the feasibility and performance of current smart home services.

#### 5.3.1. Feature Extraction

Features represent the main characteristics of the original data obtained from its quantitative values. Extracting a low number of very representative features from raw sensor data is helpful to improve the accuracy of advanced processing algorithms or further information processing stages while reducing at the same time the computational cost of activity inference. After the feature extraction phase, the raw dataset is transformed into a set of features vectors, which should contain proper information to be the input of the activities discrimination and learning algorithms. The most commonly used approaches of feature extraction operate in three domains: time domain, frequency domain and discrete domain [144]. Table 6 summarizes the main features that can be extracted in each of these three domains. 

**Table 6:** Taxonomy of extracted features in three domains.

Domain	Extracted Features
Time domain	Mean, Median, Average, Variance, Standard Deviation, Minimum, Maximum, Range, Root Mean Square (RMS), Correlation, Cross-Correlation, Zero-Correlation, Integration, Differences, Velocity, Signal magnitude area (SMA), Signal vector magnitude (SVM), Difference, Zero-crossing.
Frequency domain	Wavelet Transformation, Fourier Transform (DC component, Key Coefficients, Coefficients sum, Dominant frequency, Spectrum Energy, Spectrum Entropy, Spectrum centroid)
Discrete domain	Euclidean-based Distances, Dynamic Time Warping, Levenshtein Edit Distance

Among time domain features, the mean is the most common for almost all sensor types due to its low computational cost and minimum memory requirements. The standard deviation that represents the stability of a signal around its mean is frequently used as the basic metric for classifiers or threshold-based algorithms. The median, on its side, is very useful to replace missing values from a sequence of discrete measurements.

Frequency domain features focus on the periodic structure of sensor data. Wavelet transformation is mainly used to detect the transition between different activities due to its ability to capture sudden changes in signals, especially in acceleration ones. The Fourier transform can be computed for a time-based discrete signal over a specific window length by using algorithms such as the Fast Fourier Transform (FFT) and the Fast Time Frequency Transform (FTFT). This gives information on the main frequency components present in the signal.

Discrete domain features are used to map raw sensor signals into strings of discrete symbols. Euclidean-based Distances, Dynamic Time Warping and Levenshtein Edit Distance are key approaches used to evaluate string similarity for classifying human activities and modelling behavioural patterns. 

Lara and Labrador [145] summarize the main feature extraction techniques useful for acceleration, environmental measurements and vital signs. They describe time-domain features as the ones mostly used on environmental measurements such as those obtained by binary sensors. In the case of acceleration data, time-domain and frequency-domain features are commonly used, whereas time-based features such as the number of heart beats are used on vital signals.

#### 5.3.2. Feature Selection

Features extracted from raw sensor data may contain redundant and irrelevant information, which can negatively affect system performance. Feature selection plays an effective role in selecting more discriminative features and reducing the dimensionality of feature vector. This way, the main task of the feature selection process is to find a more relevant subset of features from within a high dimensional feature vector, in order to reduce computational expense and noise, and to benefit the application of learning models. 

In the current literature a number of feature selection methods have been used according to the different characteristics of extracted features. Andreas *et al.* [118] used the Minimum Redundancy and Maximum Relevance (MRMR) feature selection method to choose the feature subset from the saccade, fixation, blink and wordbook feature groups, extracted from the eye movement data by using an EOG system. The same method was used in [146]. Maurer *et al.* [147] used Correlation-based Feature Selection (CFS) on an accelerometer dataset. This method is convenient since it is built into WEKA [148], an open source tool widely used for machine learning. Optimal features are selected under the assumption that these features are highly correlated with the given class and loosely correlated between different classes. Some other methods, including SVM-Based Feature Selection [149], Correlation-based Feature Selection [150], Sequential Forward Floating Search [151], and Forward-Backward Sequential Search [152], are also used to select the most relevant features from a high dimensional feature vector. Principal Component Analysis (PCA), Independent Component Analysis (ICA) and Linear Discriminant Analysis (LDA) [153] are used to map the high dimensional feature vector into a lower dimensional one. As a summary, we have compiled into Table 7 the correspondences found in relevant literature between the monitored activities, the most appropriate sensors and the associated data processing algorithms. This is a first step towards bridging the gap between the high-level description of a SH system and the lower level data management activities that are necessary to implement and deploy it.

**Table 7:** Data processing approaches correspondence with relevant sensors and monitored activities.

Activities		Sensors	Preprocessing Methods	Segmentation Methods	Dimensionality Reduction Methods
BADLs	Bathing, dressing, eating and drinking, using toilet, grooming	- Contact switches [72] - Accelerometer, temperature sensor, altimeter [63] - Presence sensors [113]	- WMA technique [63] - Median filter [113]	- Sliding window [63] - Sliding window 5 s long [113] - Sensor event-based similarity measure [70,84]	- Sensor fires within time interval, sensor fires before another sensor within time interval [72] - Mean, min, max, standard deviation, variance, difference, range, root-mean-square, correlation, spectral energy, spectral entropy, key coefficient [63]
IADLs	Using telephone, watching TV, preparing food, cleaning, taking medicine, sleeping	- Accelerometer, temperature sensor, altimeter, gyroscope, light sensor, heart rate sensor, barometer [37] - Presence sensors [112] - Motion sensors, contact switches [89] - Accelerometer, audio recorder, RFID [94] - Altimeter, microphone, presence sensors, door contacts, temperature sensors [60]	- Undersampling [37] - Median filter and FOH filter [112] - Bayes filter and particle filter [89] - Low-pass filter [94]	- Sliding window [37] - Temporal- based window [94,112] - Sensor event-based similarity measure [70,84]	- Mean, min, max, standard deviation, median, mode, kurtosis, skewness, intensity, difference, energy, entropy, root-mean-square, correlation, key coefficient [37] - Percentage of time spent in various postures, number of events per class, percentage of time in each room, percentage of time “open” and predominant position in the time frame [60] - DC mean, variance, energy, frequency-domain entropy and correlation for acceleration data; standard deviation, range and zero-crossing rate for audio data; object name or location name for RFID reading [94]
Ambulatory activities	Lying, sitting, standing, walking, running, cycling, walking upstairs, walking downstairs, lie-to-stand, stand-to-lie, sit-to-stand, stand-to sit	- Accelerometer [96,108,109,113,114,115,140] - Accelerometer and gyroscope [32,101]	- High-pass filter [101] - Median filter, Kalman filter, low-pass filter and discrete wavelet package shrinkage [115] - Low-pass filter [114] - Linear interpolation [109]	- Sliding window [113] - Temporal-based window [96] - Activity-based window [140] - SWAB [109]	- Mean, min, max, root mean square, standard deviation [113] - 42-dimensional time domain feature, FFT and DCT coefficients [114] - Sum, mean, standard deviation, coefficient, peak-to-peak amplitude, range, correlation, zero crossing [96] - Mean and standard deviation of acceleration and angular speed [32] - Discrete Cosine Transform and PCA [140] - Mean, standard derivation, derivation, zero or mean crossing rate, root mean square, peak count, spectral energy, spectral entropy, spectral centroid [108] - Discrete Wavelet Transform [101] - Euclidean-based Distances, Dynamic Time Warping [109]
Vital sign monitoring	Brain activity and heart rate	- ECG [76,116,153] - EEG, ECG [42,110]	- Extended Kalman filter [116] - 8 band-pass filters [42] - Low-pass filter and high-pass filter [153]	-Temporal-based window with 2 s [42]	- Spectral energy, number of peak points interval [42] - Average, Discrete-time Fourier Transformation, spectrum centroid, spectral edge frequency, mean, standard deviation, root mean square [110] - Wavelet Transform, PCA, LDA, ICA [153]
	Eye movement	EOG [117,118]	- Low-pass filter, high-pass filter [117] - Median filter, low-pass filter and wavelet package shrinkage [118]		- Mean, variance or max EOG signal amplitudes or rate of small or large, positive or negative saccades; mean or variance of the horizontal or vertical EOG amplitude within or duration of a fixation; mean or variance of the blink duration or blink rate; wordbook size or max, difference between max and min, mean or variance of all occurrence counts [118]
	Muscle activity	- EMG [119]	- Bandpass filter between 20 Hz and 450 Hz, notch filter [119]	- Sliding window [119]	- Slope sign changes, number of zero crossing, waveform length, time domain parameters, sample skewness, autoregressive model parameters; LDA [119]

## 6. Identified Research Challenges

We explore in this section the main research challenges that stem from the reviewed literature, related to the enhancement of quality of life in SH for older people.

### 6.1. Accuracy and Robustness in Activity Recognition

Some human activities are quite complex to detect either because they are composed of many lower level actions, or because these actions may be different for different people or different time. Most research on this field is more focused on recognizing what activity is taking place rather than also evaluating how correctly is the activity being carried out and other qualitative information. For example, a system can detect that the activity “dressing” is occurring, but it is much more difficult to get the information of whether the user performs this activity correctly or not. There are activities that have similar characteristics, such as “drinking milk” and “drinking water”. However, the nutritional implications of them are very different. In order to enhance the discrimination of similar activities several researchers have tried to add more sensors to add accuracy to the activity monitoring. 

In addition to the above, there are many other factors that can affect the accuracy and robustness of the activity monitoring. For example, an activity can be performed in different ways by different users, or even by the same user in different time periods. Therefore, the robustness of the system to recognize the activity becomes a challenge. For addressing this issue, some researchers have tried to train algorithms with a larger amount of sensor data to capture more features. Furthermore, a variety of wearable devices have been developed to achieve real-time full-body activity monitoring. In summary a clear understanding of what activity is being performed by the user in terms of accuracy and robustness is still highly challenging. 

### 6.2. High-Level and Long-Term Activity Monitoring

Most current work on activity monitoring and detection focuses on relatively short low-level activities that are performed in seconds or minutes in laboratory environments. The monitoring of high-level activities on larger time scales and in real-world scenarios is clearly more difficult in terms of the obtention of the data and proper formalization of the activities. Yet, these high-level long-term real activities monitoring is key for detecting behavioural patterns of elderly people. 

At this respect there is a lack of formalization proposals for high-level activities and their required sensors. Normally these activities contain several sub-activities that may be performed in different order or even in parallel. It would be highly beneficial to define a high-level language for describing activities that contain other activities with high flexibility. In addition, monitoring these activities requires more seamless and efficient data gathering and processing techniques to maximize users’ acceptance and minimize the burden posed on people. Making sensors more robust, less obtrusive and more comfortable to wear also becomes a challenge when dealing with high-level and long-term activities monitoring.

### 6.3. Multi-User and Multi-Sensor Activity Monitoring

In most recent experiments, sensor data is collected and analysed from a single user activity in smart home. However, in real life scenarios, activities can be performed by multiple users concurrently and there may be interaction between them. Activity monitoring of multiple users is clearly more challenging. The issues to be addressed include the design of appropriate sensors to capture the interaction between multiple users, finding suitable methods to model this interaction, and inferring useful information from these observations.

Not only multiple users, but also multiple sensors pose research challenges. To detect most activities a single sensor cannot provide with enough information. Some works have been done to embed diverse sensors in smart phones in order to promote applications for human movement monitoring. Anyway, even if the physical sensors are already available, to process different sensor data in order to provide sufficient accuracy for activity monitoring is still an open research issue.

### 6.4. Real World Data Collection

On many occasions elderly people activity monitoring is performed in laboratory environments. In these cases, the experts design controlled experiments and the activities to be carried out depending on the requirements of the research. Participants perform the activities following the experts’ guidelines. For example, when detecting fall situations for elderly people, participants fall in a controlled manner as specified for each devised scenario.

These experiments are helpful for experts to collect data and choose the appropriate methods to process it, but in a real world scenario it is almost impossible to foresee the circumstances under which some activities will occur. Moreover, elderly people may perform two or three activities at the same time (such as drinking water while watching TV, short hand gestures while jogging and walking) and one activity may be interrupted by another one (such as answering the telephone while preparing a meal). Thus it is still a challenge to apply the algorithms to real world activity monitoring. There is also a lack of benchmarks containing annotated reference datasets for researchers to apply, assess and compare the results of their proposed approaches.

### 6.5. Heterogeneous Sensor Data Representation

Generally, the data collected from sensors in smart environments are heterogeneous in format, structure and semantics, especially when multimodal sensors and different types of sensor are used to obtain the raw data. These datasets are difficult to share and reuse due to the lack of formalised descriptions of them. Some researchers have tried to develop high-level descriptive sensor data models to address these problems. These models are used to support semantic sensor data management and intelligent processing. In the future, knowledge descriptive sensor data modelling and standardization are needed to advance towards a commonly accepted framework for heterogeneous sensor data representation.

### 6.6. Imbalanced and Overlapping Data Classes 

During the phase of processing sensor data, researchers usually encounter the problems of imbalanced class distribution and class overlapping, especially in real-time scenarios. For instance, when performing long-time monitoring for elderly people with diabetes, sleeping, eating and drinking occur frequently, but walking may occur less frequently. The class imbalance problem occurs when the number of instances of one class (minority class) is extremely low compared to other ones (majority class). This problem often exits when multiple data classes are defined and multiple sensors datasets exist. The class overlapping problem means that the characteristics of the examples for training algorithms are very similar for two or more classes, rendering it difficult to distinguish between these classes. 

Imbalanced class and overlapping class issues have great impact on classification tasks and recognition accuracy. To deal with this issue, some researchers have adopted sampling-based algorithms as part of the data preprocessing in order to level majority and minority classes. A balanced dataset is obtained by oversampling the minority class (duplicating some of the available samples) and undersampling the majority class (discarding redundant samples). However, this method may result in useful information being lost. Therefore, determining a proper class distribution is still a problem to be solved for human activity monitoring and modelling in real world environments.

### 6.7. Meaningful Feature Extraction 

As previously described, the automatic detection of activities from raw sensor data needs proper preprocessing methods and feature extraction. The preprocessing methods mainly focus on eliminating noise from the information (cleaning, completing and normalizing data). Feature extraction aims on one hand to choose the essential features that are relevant for the activity detection task and, on the other hand, to support dimensionality reduction for more robust modelling and processing. Current feature extraction approaches are normally based on time or frequency analysis. However, if it were possible to have an approach based on heuristics obtained from a deep understanding of the activities, better accuracy and robustness would be obtained since meaningful criteria would be considered. A more thorough research on proper feature extraction methods based on the nature of human activities could be a promising future research trend.

### 6.8. Consideration of Human Factors

E-Health solutions are generally regarded as socio-technical systems since, in addition to its technological nature, many people interact with them during the provision of the service. Thus, the service must meet the expectations and needs of all the involved people, if it has to be of any practical use. Human factors directly affect the acceptance of the service by these people. 

Although efforts have been made to identify and formalize the different areas that conform the human factors, these have not been yet adequately addressed from a formal point of view. Besides, human factors are by nature technically difficult to address. In many cases their proper consideration requires a good understanding of not only people’s capabilities and limitations but also their personal, socioeconomic and cultural context. Even with this understanding, it is often difficult to translate it into the formal terms required by the information and communication technologies to be used inside SH. All this hinders research and innovation when developing new solutions.

To address this issue research initiatives are required which are aimed at creating design patterns and low level guidelines to promote the integration of the human factors-related functional aspects. These would constitute a set of very useful resources for developers which, if properly characterized, could be easily particularized to each specific application, person and context.

## 7. Conclusions

We have presented a review of the main concepts, devices, techniques and models used for the provision of services tailored to enhance independent living for the elderly in smart homes. This review is aimed at making it easier for developers and service providers to construct and deploy complete solutions. To achieve this, we have presented the review in a top-down approach, beginning with the services provider vision. This way we have started with the services perspective, gone through high-level concepts such as the activities, and finished with the description of sensors and the processing of their data. In each of the sections we have paid special attention to the issues more related to the provision of real services.

The taxonomy of activities that we have presented allows one to make the development of services for the elderly independent of the final home devices configuration. This developer-centered approach permits, as a plus, to specify solutions which are adequate for a wider range of final users. To do so, we have comprehensively reviewed the main activities related to the elderly’s health found in the literature, and classified them into types. This way, it also becomes easier to generalize the data processing techniques that have to be applied for the detection of the activities.

Besides the activities classification, a review on the main approaches for their formalized description has been conducted. If an adequate formalization is used, the services and the infrastructure may be specified in a decoupled manner. We have not only presented the principal formalization techniques, but also identified their potential shortcomings when applied to real deployments. One of the most significant limitations found is related to the modelling and characterization of the relationships between different activities. This is a critical issue because in real life it is uncommon to find activities that are totally unrelated to others. Moreover, a correct identification of the activities that are related to a given one may greatly enhance the precision of its detection. At this respect, we have proposed a first general schema which has to be further developed in the future.

Based on the analysis made of the activities, we have described a classification of sensors that can be deployed in smart homes, and summarized the data processing techniques applicable to the sensor data collected from them. We have also presented a discussion of the advantages as well as the disadvantages of the different sensors and their data processing techniques, to aid the researchers in the selection of the proper methods according to the requirements.

Another relevant aspect of the presented work is the identification of some important research challenges that should be tackled to enhance the performance, precision and robustness of the systems considered. We hope that this description contributes to the definition of future research projects and the subsequent advancement in this knowledge area.

After reviewing the relevant literature, and also considering our expertise in the development of smart home solutions, we have come to the conviction that the precision when modelling the sensors infrastructure together with their activity detection capacities is a crucial factor. Unlike other types of systems, embedding technology into a home is critical because it raises important privacy issues. The acceptance of the solutions inside the home will strongly depend on the correct adjustment to the users’ necessities and the perceived effects they have on people’s daily lives. If a correct characterization of these human factors is achieved from the very beginning of the solution development, it is much more likely for it to succeed.

In close relation to the above, we find it crucial to correctly characterize and formalize the activities carried out inside home in order to deploy useful final services. If a correct understanding exists on the relationship between the deployed sensors and their activity detection possibilities, the design of the solution and its deployment could be greatly enhanced and done more easily and faster.

As a last remark we highlight that, in most reviewed work, research teams have created ideal conditions for the activity detection. These results are obviously interesting and add to the knowledge in this area, however they should be considered as base elements with which a more advanced support for people in real environments could be built and provided. The presence of more than one person interacting in the same place, as well as the coexistence of interrelated activities, pose true challenges to be studied in the following years.

## References

[1] Lutolf R. Smart home concept and the integration of energy meters into a home based system. Proceedings of the 7th International Conference on Metering Apparatus and Tariffs for Electricity Supply.

[2] Satpathy L. (2006). Smart Housing: Technology to Aid Aging in Place, New Opportunities and Challenges. Master’s Thesis.

[3] 10 Facts on Ageing and the Life Course. http://www.who.int/features/factfiles/ageing/en/.

[4] Giannakouris K. (2008). Ageing characterises the demographic perspectives of the european societies. Stat. Foc..

[5] Lloret J., Canovas A., Sendra S., Parra L. (2015). A smart communication architecture for ambient assisted living. IEEE Commun. Mag..

[6] Aghajan H., Augusto J.C., Wu C., McCullagh P., Walkden J.-A. (2007). Distributed vision-based accident management for assisted living. Pervasive Computing for Quality of Life Enhancement.

[7] Ali R., Siddiqi M.H., Lee S., Kang B.H. A hybrid reasoning approach for promoting active lifestyle. Proceedings of the 9th International Conference on Ubiquitous Information Management and Communication.

[8] Alam M.R., Reaz M.B.I., Ali M.A.M. (2012). A review of smart homes—Past, present, and future. IEEE Trans. Syst. Man Cybern. C Appl. Rev..

[9] Rashidi P., Mihailidis A. (2013). A survey on ambient-assisted living tools for older adults. IEEE J. Biomed. Health Inform..

[10] Salih A., Abraham A. (2013). A review of ambient intelligence assisted healthcare monitoring. IJCISIM.

[11] Peetoom K.K., Lexis M.A., Joore M., Dirksen C.D., De Witte L.P. (2014). Literature review on monitoring technologies and their outcomes in independently living elderly people. Disabil. Rehabil. Assist. Technol..

[12] Khusainov R., Azzi D., Achumba I.E., Bersch S.D. (2013). Real-time human ambulation, activity, and physiological monitoring: Taxonomy of issues, techniques, applications, challenges and limitations. Sensors.

[13] Avci A., Bosch S., Marin-Perianu M., Marin-Perianu R., Havinga P. Activity recognition using inertial sensing for healthcare, wellbeing and sports applications: A survey. Proceedings of the 23rd international conference on Architecture of computing systems (ARCS).

[14] Bulling A., Blanke U., Schiele B. (2014). A tutorial on human activity recognition using body-worn inertial sensors. ACM Comput. Surv..

[15] Helal S., Mann W., El-Zabadani H., King J., Kaddoura Y., Jansen E. (2005). The gator tech smart house: A programmable pervasive space. Computer.

[16] Cook D.J., Youngblood M., Heierman E.O., Gopalratnam K., Rao S., Litvin A., Khawaja F. Mavhome: An agent-based smart home. Proceedings of the IEEE International Conference on Pervasive Computing and Communications (PerCom).

[17] Logan B., Healey J., Philipose M., Tapia E.M., Intille S. (2007). A long-term evaluation of sensing modalities for activity recognition. Ubiquitous Computing.

[18] Van Kasteren T.L.M., Englebienne G., Kröse B.J.A. (2010). Activity recognition using semi-markov models on real world smart home datasets. J. Ambient Intell. Smart Environ.

[19] Chen C., Dawadi P. Casasviz: Web-based visualization of behavior patterns in smart environments. Proceedings of the International Conference on Pervasive Computing and Communications Workshops (PERCOM Workshops).

[20] Cook D.J., Crandall A.S., Thomas B.L., Krishnan N.C. (2013). CASAS: A smart home in a box. Computer.

[21] Vacher M., Istrate D., Portet F., Joubert T., Chevalier T., Smidtas S., Meillon B., Lecouteux B., Sehili M., Chahuara P. The sweet-home project: Audio technology in smart homes to improve well-being and reliance. Proceedings of the Annual International Conference on Engineering in Medicine and Biology Society.

[22] Chahuara P., Portet F., Vacher M. (2014). Making context aware decision from uncertain information in a smart home: A markov logic network approach. Ambient Intelligence.

[23] Antoniou P.E., Konstantinidis E.I., Billis A.S., Bamidis P.D. (2014). Integrating the usefil assisted living platform; observation from the field. Proceedings of the 6th European Conference of the International Federation for Medical and Biological Engineering.

[24] Billis A.S., Papageorgiou E.I., Frantzidis C., Konstantinidis E.I., Bamidis P.D. Towards a hierarchically-structured decision support tool for improving seniors’ independent living: The usefil decision support system. Proceedings of the 6th International Conference on PErvasive Technologies Related to Assistive Environments.

[25] Zhang Q., Karunanithi M., Rana R., Liu J. Determination of activities of daily living of independent living older people using environmentally placed sensors. Proceedings of the 35th Annual International Conference on Engineering in Medicine and Biology Society (EMBC).

[26] Zhang Q., Su Y., Yu P. (2014). Assisting an elderly with early dementia using wireless sensors data in smarter safer home. Service Science and Knowledge Innovation.

[27] List of Home Datasets. http://boxlab.wikispaces.com/List+of+Home+Datasets.

[28] Roggen D., Calatroni A., Rossi M., Holleczek T., Forster K., Troster G., Lukowicz P., Bannach D., Pirkl G., Ferscha A. Collecting complex activity datasets in highly rich networked sensor environments. Proceedings of the 7th International Conference on Networked Sensing Systems (INSS).

[29] Baños O., Damas M., Pomares H., Rojas I., Tóth M.A., Amft O. A benchmark dataset to evaluate sensor displacement in activity recognition. Proceedings of the 2012 ACM Conference on Ubiquitous Computing.

[30] Pulkkinen T., Son Y.-S., Lee J., Lee Y.-H., Sallinen M., Park J.-H. Progressive monitoring and treatment planning of diabetes mellitus in smart home environment. Proceedings of the International Conference on Consumer Electronics (ICCE).

[31] Chatterjee S., Dutta K., Xie H., Byun J., Pottathil A., Moore M. Persuasive and pervasive sensing: A new frontier to monitor, track and assist older adults suffering from type-2 diabetes. Proceedings of the 46th Hawaii International Conference on System Sciences (HICSS).

[32] Chiang S.Y., Kan Y.C., Tu Y.C., Lin H.C. (2013). A preliminary activity recognition of wsn data on ubiquitous health care for physical therapy. Recent Progress in Data Engineering and Internet Technology.

[33] Sardini E., Serpelloni M. (2014). T-shirt for vital parameter monitoring. Sensors.

[34] Honeywell Genesis Series Cable. http://www.honeywellcable.com.

[35] Telestation-Philips Telehealth. http://www.telehealth.philips.com.

[36] Bosch Healthcare. http://www.bosch-telehealth.com.

[37] Chernbumroong S., Cang S., Yu H. (2014). A practical multi-sensor activity recognition system for home-based care. Decis. Support Syst..

[38] Lara O.D., Pérez A.J., Labrador M.A., Posada J.D. (2012). Centinela: A human activity recognition system based on acceleration and vital sign data. Pervasive Mob. Comput..

[39] Chaminda H.T., Klyuev V., Naruse K. A smart reminder system for complex human activities. Proceedings of the 14th International Conference on Advanced Communication Technology (ICACT).

[40] Hussain M., Ali T., Khan W.A., Afzal M., Lee S., Latif K. (2015). Recommendations service for chronic disease patient in multimodel sensors home environment. Telemed. E Health.

[41] Suryadevara N.K., Mukhopadhyay S.C., Wang R., Rayudu R.K. (2013). Forecasting the behavior of an elderly using wireless sensors data in a smart home. Eng. Appl. Artif. Intel..

[42] Han Y., Han M., Lee S., Sarkar A.M., Lee Y.-K. (2012). A framework for supervising lifestyle diseases using long-term activity monitoring. Sensors.

[43] Chandola V., Banerjee A., Kumar V. (2009). Anomaly detection: A survey. ACM Comput. Surv..

[44] Phua C., Foo V.-F., Biswas J., Tolstikov A., Maniyeri J., Huang W., That M.-H., Xu D., Chu A.-W. 2-layer erroneous-plan recognition for dementia patients in smart homes. Proceedings of the 11th International Conference on e-Health Networking, Applications and Services.

[45] Lotfi A., Langensiepen C., Mahmoud S.M., Akhlaghinia M.J. (2012). Smart homes for the elderly dementia sufferers: Identification and prediction of abnormal behaviour. J. Ambient Intell. Humaniz. Comput..

[46] Vogt J. (2013). Requirements Elicitation and System Specification of Assistive Systems for People with Mild Dementia. Ph.D. Thesis.

[47] Riboni D., Bettini C., Civitarese G., Janjua Z.H., Helaoui R. (2015). Extended report: Fine-grained recognition of abnormal behaviors for early detection of mild cognitive impairment. http://arxiv.org/abs/1501.05581.

[48] Ordóñez F.J., de Toledo P., Sanchis A. (2014). Sensor-based bayesian detection of anomalous living patterns in a home setting. Pers. Ubiquit. Comput..

[49] Gjoreski H., Rashkovska A., Kozina S., Lustrek M., Gams M. Telehealth using ecg sensor and accelerometer. Proceedings of the 37th International Convention on Information and Communication Technology, Electronics and Microelectronics (MIPRO).

[50] Wu G.E., Xue S. (2008). Portable preimpact fall detector with inertial sensors. IEEE Trans. Neural Syst. Rehabil. Eng..

[51] Rimminen H., Lindstrom J., Linnavuo M., Sepponen R. (2010). Detection of falls among the elderly by a floor sensor using the electric near field. IEEE Trans. Inf. Technol. Biomed..

[52] Zhuang X., Huang J., Potamianos G., Hasegawa-Johnson M. Acoustic fall detection using gaussian mixture models and gmm supervectors. Proceedings of the IEEE International Conference on Acoustics, Speech and Signal Processing.

[53] Ghasemzadeh H., Jafari R., Prabhakaran B. (2010). A body sensor network with electromyogram and inertial sensors: Multimodal interpretation of muscular activities. IEEE Trans. Inf. Technol. Biomed..

[54] Coughlin J.F., D’Ambrosio L.A., Reimer B., Pratt M.R. Older adult perceptions of smart home technologies: Implications for research, policy & market innovations in healthcare. Proceedings of the 29th Annual International Conference of Engineering in Medicine and Biology Society.

[55] Gaul S., Ziefle M. (2009). Smart home technologies: Insights into generation-specific acceptance motives. HCI and Usability for e-Inclusion.

[56] Niman B.V., Rodríguez-Ascaso A., Brown S., Sund T. (2007). User experience design guidelines for telecare (e-health) services. Interactions.

[57] Becker M. Software Architecture Trends and Promising Technology for Ambient Assisted Living Systems. http://www.researchgate.net/profile/Martin_Becker3/publication/30815935_Software_Architecture_Trends_and_Promising_Technology_for_Ambient_Assisted_Living_Systems/links/02e7e5252637478996000000.pdf.

[58] Vega-Barbas M., Pau I., Martín-Ruiz M.L., Seoane F. (2015). Adaptive software architecture based on confident hci for the deployment of sensitive services in smart homes. Sensors.

[59] MedicineNet. http://www.medicinenet.com.

[60] Fleury A., Vacher M., Noury N. (2010). SVM-based multimodal classification of activities of daily living in health smart homes: Sensors, algorithms, and first experimental results. IEEE Trans. Inf. Technol. Biomed..

[61] Katz S., Akpom C.A. (1976). A measure of primary sociobiological functions. Int. J. Health Serv..

[62] Lawton M.P., Brody E.M. (1970). Assessment of older people: Self-maintaining and instrumental activities of daily living. Nurs. Res..

[63] Chernbumroong S., Cang S., Atkins A., Yu H. (2013). Elderly activities recognition and classification for applications in assisted living. Expert Syst. Appl..

[64] Tartarisco G., Baldus G., Corda D., Raso R., Arnao A., Ferro M., Gaggioli A., Pioggia G. (2012). Personal health system architecture for stress monitoring and support to clinical decisions. Comput. Commun..

[65] Okeyo G., Chen L., Wang H. (2014). Combining ontological and temporal formalisms for composite activity modelling and recognition in smart homes. Future Gener. Comput. Syst..

[66] Meditskos G., Dasiopoulou S., Kompatsiaris I. (2015). MetaQ: A knowledge-driven framework for context-aware activity recognition combining SPARQL and OWL 2 activity patterns. Pervasive Mob. Comput..

[67] Allen J.F. (1983). Maintaining knowledge about temporal intervals. ACM Commun..

[68] Wongpatikaseree K., Ikeda M., Buranarach M., Supnithi T., Lim A.O., Tan Y. Activity recognition using context-aware infrastructure ontology in smart home domain. Proceedings of the 2012 Seventh International Conference on Knowledge, Information and Creativity Support Systems (KICSS).

[69] Chen L., Nugent C. (2009). Ontology-based activity recognition in intelligent pervasive environments. Int. J. Web Inf. Syst..

[70] Ye J., Stevenson G., Dobson S. (2015). KCAR: A knowledge-driven approach for concurrent activity recognition. Pervasive Mob. Comput..

[71] Cook D., Schmitter-Edgecombe M., Crandall A., Sanders C., Thomas B. Collecting and disseminating smart home sensor data in the casas project. Proceedings of the CHI Workshop on Developing Shared Home Behavior Datasets to Advance HCI and Ubiquitous Computing Research.

[72] Tapia E.M., Intille S.S., Larson K. (2004). Activity recognition in the home using simple and ubiquitous sensors. Pervasive Computing.

[73] Aiello F., Fortino G., Gravina R., Guerrieri A. (2011). A java-based agent platform for programming wireless sensor networks. Comput. J..

[74] World Wide Web Consortium Composite Capability/Preference Profiles (CC/PP): Structure and Vocabularies 1.0. http://www.w3.org/TR/2004/REC-CCPP-struct-vocab-20040115.

[75] McDonald H., Nugent C.D., Finlay D.D., Moore G., Burns W., Hallberg J. (2013). Assessing the impact of the homeml format and the homeml suite within the research community. J. UCS.

[76] Gonçalves B., Pereira Filho J.G., Andreão R.V. ECGWARE: An ECG Markup Language for Ambulatory Telemonitoring and Decision Making Support. Proceedings of the International Conference on Health Informatics.

[77] Rialle V., Lamy J.-B., Noury N., Bajolle L. (2003). Telemonitoring of patients at home: A software agent approach. Comput. Meth. Prog. Bio..

[78] Henricksen K., Indulska J. A software engineering framework for context-aware pervasive computing. Proceedings of the Second IEEE Annual Conference on Pervasive Computing and Communications.

[79] Perera C., Zaslavsky A., Christen P., Georgakopoulos D. (2014). Context aware computing for the internet of things: A survey. IEEE Commun. Surv. Tut..

[80] Zhang D., Gu T., Wang X. (2005). Enabling context-aware smart home with semantic web technologies. Int. J. Hum. Welf. Robotic Syst..

[81] Bouchard B., Giroux S., Bouzouane A. (2006). A smart home agent for plan recognition of cognitively-impaired patients. J. Comput..

[82] Chen L., Nugent C., Mulvenna M., Finlay D., Hong X., Poland M. (2008). Using event calculus for behaviour reasoning and assistance in a smart home. Smart Homes and Health Telematics.

[83] Chen H., Perich F., Finin T., Joshi A. SOUPA: Standard ontology for ubiquitous and pervasive applications. Proceedings of the First Annual International Conference on Mobile and Ubiquitous Systems: Networking and Services.

[84] Ye J., Stevenson G., Dobson S. (2014). USMART: An unsupervised semantic mining activity recognition technique. ACM Trans. Inter. Intel. Syst..

[85] Chen L., Nugent C.D., Wang H. (2012). A knowledge-driven approach to activity recognition in smart homes. IEEE Trans. Knowl. Data Eng..

[86] Musen M.A. (2013). Protégé ontology editor. Encyclopedia of Systems Biology.

[87] Rialle V., Ollivet C., Guigui C., Hervé C. (2009). What do family caregivers of Alzheimer’s disease patients desire in smart home technologies?. http://arxiv.org/ftp/arxiv/papers/0904/0904.0437.pdf.

[88] Portet F., Vacher M., Golanski C., Roux C., Meillon B. (2013). Design and evaluation of a smart home voice interface for the elderly: Acceptability and objection aspects. Pers. Ubiquitous Comput..

[89] Wilson D.H., Atkeson C. (2005). Simultaneous tracking and activity recognition (STAR) using many anonymous, binary sensors. Pervasive Computing.

[90] Ordónez F.J., de Toledo P., Sanchis A. (2013). Activity recognition using hybrid generative/discriminative models on home environments using binary sensors. Sensors.

[91] Fujinami T., Miura M., Takatsuka R., Sugihara T. (2011). A study of long term tendencies in residents’ activities of daily living at a group home for people with dementia using rfid slippers. Toward Useful Services for Elderly and People with Disabilities.

[92] Philipose M., Fishkin K.P., Perkowitz M., Patterson D.J., Fox D., Kautz H., Hahnel D. (2004). Inferring activities from interactions with objects. IEEE Pervasive Comput..

[93] Kim S.-C., Jeong Y.-S., Park S.-O. (2013). RFID-based indoor location tracking to ensure the safety of the elderly in smart home environments. Pers. Ubiquitous Comput..

[94] Wang L., Gu T., Tao X., Chen H., Lu J. (2011). Recognizing multi-user activities using wearable sensors in a smart home. Pervasive Mob. Comput..

[95] Zhu C., Sheng W. (2011). Motion-and location-based online human daily activity recognition. Pervasive Mob. Comput..

[96] Zheng Y., Wong W.K., Guan X., Trost S. Physical activity recognition from accelerometer data using a multi-scale ensemble method. Proceedings of the 25th Conference on Innovative Applications of Artificial Intelligence.

[97] Gao L., Bourke A.K., Nelson J. (2014). Evaluation of accelerometer based multi-sensor *versus* single-sensor activity recognition systems. Med. Eng. Phys..

[98] Narayanan M.R., Redmond S.J., Scalzi M.E., Lord S.R., Celler B.G., Lovell N.H. (2010). Longitudinal falls-risk estimation using triaxial accelerometry. IEEE Trans. Biomed. Eng..

[99] Greene B.R., Donovan A.O., Romero-Ortuno R., Cogan L., Scanaill C.N., Kenny R.A. (2010). Quantitative falls risk assessment using the timed up and go test. IEEE Trans. Biomed. Eng..

[100] Zijlstra W., Bisseling R.W., Schlumbohm S., Baldus H. (2010). A body-fixed-sensor-based analysis of power during sit-to-stand movements. Gait Posture.

[101] Kau L.-J., Chen C.-S. A smart phone-based pocket fall accident detection system. Proceedings of the 2014 IEEE International Symposium on Bioelectronics and Bioinformatics (ISBB).

[102] Varkey J.P., Pompili D., Walls T.A. (2012). Human motion recognition using a wireless sensor-based wearable system. Pers. Ubiquitous Comput..

[103] Gjoreski H., Gams M. Accelerometer data preparation for activity recognition. Proceedings of the International Multiconference Information Society.

[104] Jiang M., Shang H., Wang Z., Li H., Wang Y. (2011). A method to deal with installation errors of wearable accelerometers for human activity recognition. Physiol. Meas..

[105] Kwapisz J.R., Weiss G.M., Moore S.A. (2011). Activity recognition using cell phone accelerometers. ACM SIGKDD Explor..

[106] Siirtola P., Röning J. (2012). User-independent human activity recognition *vs.* Real-time on device recognition. Distributed Computing and Artificial Intelligence.

[107] Hemalatha C.S., Vaidehi V. (2013). Frequent bit pattern mining over tri-axial accelerometer data streams for recognizing human activities and detecting fall. Proc. Comput. Sci..

[108] Mannini A., Intille S.S., Rosenberger M., Sabatini A.M., Haskell W. (2013). Activity recognition using a single accelerometer placed at the wrist or ankle. Med. Sci. Sports Exerc..

[109] Muaaz M., Mayrhofer R. An analysis of different approaches to gait recognition using cell phone based accelerometers. Proceedings of the 11th International Conference on Advances in Mobile Computing & Multimedia.

[110] Ou Y.-Y., Hsia C.-C., Wang J.-F., Kuan T.-W., Hsieh C.-H. SVM-based IADL score correlation and classification with EEG/ECG signals. Proceedings of the 2013 International Conference on Orange Technologies (ICOT).

[111] Matthews R., McDonald N.J., Hervieux P., Turner P.J., Steindorf M.A. A wearable physiological sensor suite for unobtrusive monitoring of physiological and cognitive state. Proceedings of the 29th Annual International Conference of the IEEE Engineering in Medicine and Biology Society.

[112] Noury N., Hadidi T. (2012). Computer simulation of the activity of the elderly person living independently in a health smart home. Comput. Meth. Prog. Biomed..

[113] Guettari T., Boudy J., Benkelfat B.E., Chollet G., Baldinger J.L., Dore P., Istrate D. Thermal signal analysis in smart home environment for detecting a human presence. Proceedings of the 1st International Conference on Advanced Technologies for Signal and Image Processing (ATSIP).

[114] Khan A.M., Siddiqi M.H., Lee S.-W. (2013). Exploratory data analysis of acceleration signals to select light-weight and accurate features for real-time activity recognition on smartphones. Sensors.

[115] Wang W.-Z., Guo Y.-W., Huang B.-Y., Zhao G.-R., Liu B.-Q., Wang L. Analysis of filtering methods for 3D acceleration signals in body sensor network. Proceedings of the International Symposium on Bioelectronics and Bioinformatics (ISBB).

[116] Sen N., Chandrakar C. (2014). Development of a novel ECG signal denoising system using extended kalman filter. Development.

[117] Champaty B., Jose J., Pal K., Thirugnanam A. Development of eog based human machine interface control system for motorized wheelchair. Proceedings of the 2014 Annual International Conference on Emerging Research Areas: Magnetics, Machines and Drives (AICERA/iCMMD).

[118] Bulling A., Ward J.A., Gellersen H., Troster G. (2011). Eye movement analysis for activity recognition using electrooculography. IEEE Trans. Pattern Anal. Mach. Intell..

[119] Khushaba R.N., Kodagoda S., Takruri M., Dissanayake G. (2012). Toward improved control of prosthetic fingers using surface electromyogram (EMG) signals. Expert Syst. Appl..

[120] Das B., Krishnan N.C., Cook D.J. (2014). Handling imbalanced and overlapping classes in smart environments prompting datase. Data Mining for Service.

[121] Goodwin M.S., Intille S.S., Albinali F., Velicer W.F. (2011). Automated detection of stereotypical motor movements. J. Autism Dev. Disord..

[122] Parlak S., Marsic I. (2013). Detecting object motion using passive RFID: A trauma resuscitation case study. IEEE Trans. Instrum. Meas..

[123] Shayei A., Ehsani S.P., Shabany M. Efficient implementation of real-time ecg derived respiration system using cubic spline interpolation. Proceedings of the 2013 IEEE International Symposium on Circuits and Systems (ISCAS).

[124] Rodner T., Litz L. Data-driven of rule-based behavior models for an ambient assisted living system. Proceedings of the IEEE 3rd International Conference on Consumer Electronics.

[125] Sun X., Zhang Y. Design and implementation of portable ecg and body temperature monitor, Computer. Proceedings of the 2014 International Symposium on Consumer and Control (IS3C).

[126] Mitchell H.B. (2007). Sensor value normalization. Multi-Sensor Data Fusion.

[127] Ordóñez F.J., Iglesias J.A., de Toledo P., Ledezma A., Sanchis A. (2013). Online activity recognition using evolving classifiers. Expert Syst. Appl..

[128] Krishnan N.C., Panchanathan S. Analysis of low resolution accelerometer data for continuous human activity recognition. Proceedings of the 2008 IEEE International Conference on Acoustics, Speech and Signal Processing.

[129] Keogh E., Chu S., Hart D., Pazzani M. An online algorithm for segmenting time series. Proceedings of the 2001 IEEE International Conference on Data Mining.

[130] Van Kasteren T., Noulas A., Englebienne G., Kröse B. Accurate activity recognition in a home setting. Proceedings of the 10th International Conference on Ubiquitous Computing.

[131] Achumba I.E., Bersch S., Khusainov R., Azzi D., Kamalu U. On time series sensor data segmentation for fall and activity classification. Proceedings of the 14th IEEE International Conference on e-Health Networking, Applications and Services (Healthcom).

[132] Laguna J.O., Olaya A.G., Borrajo D. (2011). A dynamic sliding window approach for activity recognition. User Modeling, Adaption and Personalization.

[133] Krishnan N.C., Cook D.J. (2014). Activity recognition on streaming sensor data. Pervasive Mob. Comput..

[134] Cheng J., Amft O., Lukowicz P. (2010). Active capacitive sensing: Exploring a new wearable sensing modality for activity recognition. Pervasive Computing.

[135] Reddy S., Mun M., Burke J., Estrin D., Hansen M., Srivastava M. (2010). Using mobile phones to determine transportation modes. ACM Trans. Sens. Netw..

[136] Berchtold M., Budde M., Gordon D., Schmidtke H., Beigl M. SPAR: Service-based personal activity recognition for mobile phones. http://digisrv-1.biblio.etc.tu-bs.de:8080/docportal/servlets/MCRFileNodeServlet/DocPortal_derivate_00010205/SPAR.pdf.

[137] Banos O., Galvez J.-M., Damas M., Pomares H., Rojas I. (2014). Window size impact in human activity recognition. Sensors.

[138] Yoshizawa M., Takasaki W., Ohmura R. Parameter exploration for response time reduction in accelerometer-based activity recognition. Proceedings of the 2013 ACM conference on Pervasive and ubiquitous computing.

[139] Nyan M.N., Tay F.E.H., Seah K.H.W., Sitoh Y.Y. (2006). Classification of gait patterns in the time–frequency domain. J. Biomech..

[140] He Z., Jin L. Activity recognition from acceleration data based on discrete consine transform and SVM. Proceedings of the 2009 IEEE International Conference on Systems, Man and Cybernetics.

[141] Dernbach S., Das B., Krishnan N.C., Thomas B.L., Cook D.J. Simple and complex activity recognition through smart phones. Proceedings of the 8th International Conference on Intelligent Environments (IE).

[142] Fahim M. (2014). Evolutionary learning models for indoor and outdoor human activity recognition. Ph.D. Thesis.

[143] Hong X., Nugent C.D. (2013). Segmenting sensor data for activity monitoring in smart environments. Pers. Ubiquitous Comput..

[144] Figo D., Diniz P.C., Ferreira D.R., Cardoso J.M.P. (2010). Preprocessing techniques for context recognition from accelerometer data. Pers. Ubiquitous Comput..

[145] Lara O.D., Labrador M.A. (2013). A survey on human activity recognition using wearable sensors. IEEE Commun. Surv. Tut..

[146] Chen C., Das B., Cook D.J. A data mining framework for activity recognition in smart environments. Proceedings of the 6th International Conference on Intelligent Environments.

[147] Maurer U., Smailagic A., Siewiorek D.P., Deisher M. Activity recognition and monitoring using multiple sensors on different body positions. Proceedings of the International Workshop on Wearable and Implantable Body Sensor Networks.

[148] Waikato Environment for Knowledge Analysis. http://www.cs.waikato.ac.nz/ml/weka.

[149] Bron E., Smits M., van Swieten J., Niessen W., Klein S. (2014). Feature selection based on SVM significance maps for classification of dementia. Machine Learning in Medical Imaging.

[150] Zhang M., Sawchuk A.A. A feature selection-based framework for human activity recognition using wearable multimodal sensors. Proceedings of the 6th International Conference on Body Area Networks.

[151] Gupta P., Dallas T. (2014). Feature selection and activity recognition system using a single tri-axial accelerometer. IEEE Trans. Biomed. Eng..

[152] Muhammad S.A., Klein B.N., Van Laerhoven K., David K. (2012). A feature set evaluation for activity recognition with body-worn inertial sensors. Constructing Ambient Intelligence.

[153] Giri D., Acharya U.R., Martis R.J., Sree S.V., Lim T.-C., Ahamed T., Suri J.S. (2013). Automated diagnosis of coronary artery disease affected patients using IDA, PCA, ICA and discrete wavelet transform. Knowl. Based Syst..

